# PIRCh-seq: functional classification of non-coding RNAs associated with distinct histone modifications

**DOI:** 10.1186/s13059-019-1880-3

**Published:** 2019-12-20

**Authors:** Jingwen Fang, Qing Ma, Ci Chu, Beibei Huang, Lingjie Li, Pengfei Cai, Pedro J. Batista, Karen Erisse Martin Tolentino, Jin Xu, Rui Li, Pengcheng Du, Kun Qu, Howard Y. Chang

**Affiliations:** 1 Division of Molecular Medicine, Hefei National Laboratory for Physical Sciences at Microscale, the CAS Key Laboratory of Innate Immunity and Chronic Disease, CAS Center for Excellence in Molecular Cell Sciences, Department of Oncology of the First Affiliated Hospital, Division of Life Sciences and Medicine, University of Science and Technology of China, Hefei, 230027 China; 2 Center for Personal Dynamic Regulomes and Program in Epithelial Biology, Stanford University School of Medicine, CCSR 2155c, 269 Campus Drive, Stanford, CA 94305-5168 USA; 3 Guangdong Provincial Key Laboratory of Synthetic Genomics, Shenzhen Institute of Synthetic Biology, Shenzhen Institutes of Advanced Technology, Chinese Academy of Sciences, Shenzhen, 518055 China; 4 Present Address: Laboratory of Cell Biology, Center for Cancer Research, National Cancer Institute, National Institutes of Health, Bethesda, MD 20892 USA; 5 Howard Hughes Medical Institute, Stanford University, Stanford, CA 94305 USA

## Abstract

We develop PIRCh-seq, a method which enables a comprehensive survey of chromatin-associated RNAs in a histone modification-specific manner. We identify hundreds of chromatin-associated RNAs in several cell types with substantially less contamination by nascent transcripts. Non-coding RNAs are found enriched on chromatin and are classified into functional groups based on the patterns of their association with specific histone modifications. We find single-stranded RNA bases are more chromatin-associated, and we discover hundreds of allele-specific RNA-chromatin interactions. These results provide a unique resource to globally study the functions of chromatin-associated lncRNAs and elucidate the basic mechanisms of chromatin-RNA interactions.

## Introduction

RNAs are both the product of transcription and major regulators of the transcriptional process. In particular, long non-coding RNAs (lncRNAs) are numerous in eukaryotes and function in many cases as transcription regulators [1–3]. With the development of next-generation sequencing (NGS), tens of thousands of lncRNAs have been revealed in both murine and human genomes, and have emerged as important regulators for different biological processes [4, 5]. However, among all expressed lncRNAs, only a small subset are shown to be cell essential [6] or important for development [7] or immune responses [8]. Strategies to annotate biochemical properties of lncRNAs will be helpful to prioritize lncRNA candidates for functional analyses. Some well-studied cases have indicated that one major mechanism of lncRNAs is their ability to function through binding to histone-modifying complexes [9, 10]. LncRNAs can either recruit chromatin modifiers to regulate the chromatin states or directly regulate the process of transcription through chromosome looping to bridge distal enhancer elements to promoters [11, 12]. Thereby, a genome-wide identification of chromatin-associated lncRNAs may reveal functions and mechanisms of lncRNAs in mediating chromatin modification and regulating gene transcription.

A considerable amount of literature has been published concerning protein-RNA interactions. The advent of technologies such as RIP [13], CLIP [14], fRIP [15], and CARIP [16] has led to the discovery of multiple protein-associated RNAs, including many chromatin regulators. Conversely, nuclear extraction methods followed by RNA-seq have enabled the detection of lncRNAs which are physically associated with chromatin [17–19]. In addition, more recently reported methods like GRID-seq [20], MARGI [21], and SPRITE [22] can be used to capture pairwise RNA interactions with DNA. However, these approaches are not capable of revealing which chromatin modifications are associated with specific lncRNAs and are thus limited in the ability to elucidate their potential regulatory functions. For instance, a large number of lncRNAs are associated with Polycomb Repressive Complex 2 (PRC2), a key mammalian epigenetic regulator, to silence gene transcription by targeting its genomic loci and trimethylating histone H3 lysine 27 (H3K27me3) [23]. Therefore, lncRNAs associated with PRC2 complex may be enriched on heterochromatin regions with H3K27me3 modification. On the other hand, a new class of lncRNAs called super-lncRNAs was recently characterized. These lncRNAs target super-enhancers which have potential to regulate enhancer activities and transcription [24]. These super-lncRNAs may be enriched on euchromatin and active DNA regulatory elements with histone H3 lysine 27 acetylation (H3K27ac), H3 lysine 4 monomethylation (H3K4me1), and trimethylation (H3K4me3). Therefore, we believe it will be helpful to develop an experimental technology to distinguish different histone modification-associated lncRNAs, as well as analytical approaches to classify them and predict lncRNA functions based on their chromatin association patterns. Another technical challenge in studying chromatin-associated lncRNAs is avoiding interference from abundant nascent transcripts on chromatin. For example, results from GRID-seq [20] or MARGI [21], approaches recently developed to identify in situ global RNA interactions with DNA, contain significant amounts of nascent transcripts, making it difficult to distinguish whether the detected RNA is truly chromatin associated or merely captured during the process of transcription.

To address these questions, we developed a new method named Profiling Interacting RNAs on Chromatin followed by deep sequencing (PIRCh-seq), which enriches chromatin-associated RNAs in a histone modification-specific manner and classifies functional lncRNAs based on the patterns of their attachment to nucleosomes with specific chemical modifications. Compared to current techniques for detecting chromatin-RNA association, PIRCh-seq efficiently reduces the influence of nascent transcripts with a significantly lower number of intronic reads. Through performing PIRCh-seq with histone H3 and a number of different histone modification antibodies on different cell types, we identified cell type-specific relationships between lncRNAs and epigenetics. We found that chromatin-associated lncRNAs can be classified into six functional groups based on their association with chromatin modifications, which undergo dynamic changes with cell differentiation. In addition, we found that bases on lncRNAs attached to chromatin tend to be more single stranded in an allele-specific manner. Overall, our PIRCh-seq data provides novel insights into global functional and mechanistic studies of chromatin-associated lncRNAs.

## Results

### PIRCh-seq identifies RNA association with specific histone modifications in living cells

**Fig. 1:**
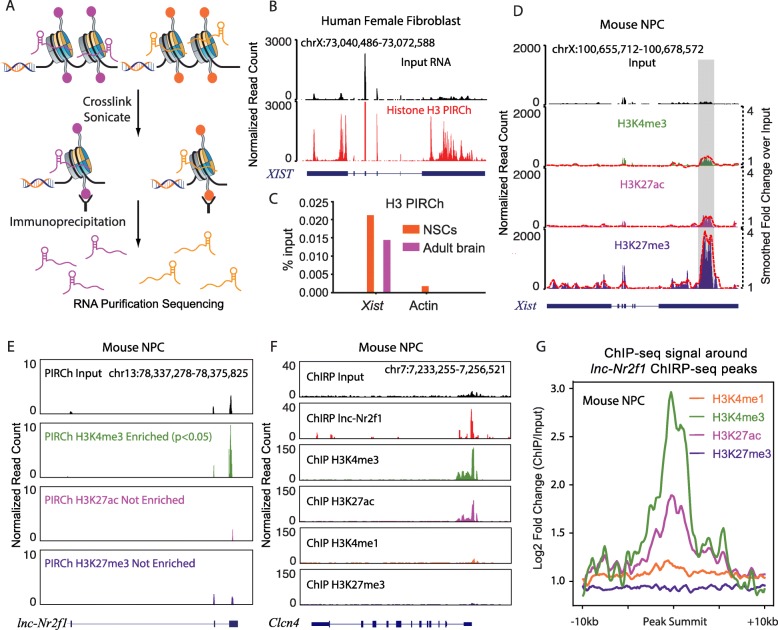
PIRCh-seq enables effective chromatin-RNA association in vivo*.* **a** Schematic representation of PIRCh approach followed by high-throughput sequencing. **b** The overall enrichment of the H3 PIRCh-seq signal (bottom) over input (top) of lncRNA *XIST* in human female fibroblasts (fold change = 19). Read counts were normalized to sequencing depth of 10 million. **c** PIRCh-qPCR analysis in mouse neuronal stem cells (NSCs, orange) and adult brain (purple) shows that *Xist* is enriched on chromatin H3 compared with Actin control. **d**, **e** Normalized input and PIRCh-seq profiles with histone modifications of H3K4me3, H3K27ac, and H3K27me3 at the lncRNA *Xist* (**d**) and *lnc-Nr2f1* (**e**) locus in mouse neuronal precursor cells (NPC). Dash lines represent fold change of PIRCh-seq over input and smoothed by 500 bp sliding windows. The boxed region represents the RepC domain on *Xist*. **f** Normalized input and ChIRP-seq profiles of lncRNA *lnc-Nr2f1* in NPC, and H3K4me3, H3K27ac, H3K4me1, and H3K27me3 ChIP-seq profiles in NPC. Showing *Clcn4* gene locus as an example. **g** Normalized log2 fold change of ChIP-seq signal over input around (± 10 kb) *lnc-Nr2f1* ChIRP-seq peaks in NPC

We conceived of PIRCh-seq as the inverse of ChIRP, a previously developed and robust method to crosslink endogenous RNA-chromatin interactions in living cells [25]. As tested by previous ChIRP-southern, 1% glutaraldehyde is better than 1% formaldehyde crosslinking in capturing RNA-chromatin associations. In the PIRCh-seq work flow, living cells are chemically crosslinked by glutaraldehyde and quenched with glycine, which prevents chromatin-associated RNA from further degradation. Chromatin is extracted and sonicated to 300–2000 base pair (bp) size, and then immunoprecipitated (IP) by histone modification-specific antibodies. Residual DNA and proteins are removed, and retrieved RNAs are then subjected to deep sequencing (the “Methods” section, Fig. 1a). We tested the possibility that glutaraldehyde crosslinking may alter the pull-down specificity of antibodies targeting histone modification. Using SNAP-ChIP [26], a pool of modified mono-nucleosomes with known histone tail modifications individually tagged with DNA barcodes, we found that glutaraldehyde crosslinking did not affect antibody specificity (Additional file 1: Figure S1A-C). The input control for PIRCh is the lysate obtained after crosslinking and sonication but not subject to IP, which was also analyzed in deep sequencing. RNAs that are retrieved by a histone modification over input beyond that expected by chance are considered PIRCh-seq hits. In this study, we generated and analyzed 26 high-resolution PIRCh-seq datasets from 2 different species—human and mouse, and 5 cell types—human H9 embryonic stem cells (H9), human female fibroblasts (HFF), mouse V6.5 embryonic stem cells (mESC), mouse embryonic fibroblasts (MEF), and mouse neuronal precursor cells (NPC), targeting histone H3 and 6 histone modifications (namely H3K4me1, H3K4me3, H3K27ac, H3K27me3, H3K9me3, and H4K16ac) and input as control with 2 replicates for each experiment (Additional file 1: Figure S1D). The correlation heatmap shows that the expressions of the input RNAs are similar to that of the total RNAs, and then from the nuclear extraction and least similar to cytoplasm (data obtained from GSE57231 and GSE32916 in the same cell line) (Additional file 1: Figure S1E), suggesting that our chosen input could serve as a reasonable baseline for chromatin-associated RNA identification. Correlation analysis of these samples indicates the high reproducibility of PIRCh-seq experiments (*R* = 0.900–0.988, Additional file 1: Figure S1F-M).

As a proof of principle, we first examined PIRCh-seq signal of the well-characterized lncRNA *XIST*, which coats the inactive X chromosome in female cells, and is known to be associated with heterochromatin with repressive histone modifications [27]. Indeed, we observed that the PIRCh-seq signal of *XIST* is highly enriched on histone H3 over input in human female fibroblast cells (Fig. 1b), as was histone H3 PIRCh followed by qRT-PCR for *Xist* in female murine neural stem cells (NSCs) and intact adult brain (Fig. 1c). These results suggest that PIRCh-seq is not only capable of enriching chromatin-associated lncRNAs, but may be applied to study brain tissue in vivo. Similarly, the lncRNA *KCNQ1OT1*, which is involved in imprinting in Beckwith-Wiedemann syndrome by silencing lineage-specific transcription through chromatin regulation [28], is also enriched on histone H3 over input, as expected (Additional file 1: Figure S2A). Additionally, the imprinted oncofetal lncRNA *H19* [29] was also enriched by histone H3 PIRCh-seq (Additional file 1: Figure S2B). On the other hand, abundant protein-coding and house-keeping mRNAs, such as *ACTB* or *EEF2*, did not show PIRCh-seq enrichment as expected for cytoplasmic mRNAs (Additional file 1: Figure S2C-D).

Next, we checked whether PIRCh-seq could enrich for RNAs associated with specific histone modifications. We performed PIRCh-seq on female NPCs with three ENCODE consortium validated antibodies targeting H3K4me3, H4K27ac, and H3K27me3. PIRCh-seq in female NPCs demonstrated that *Xist* RNA was enriched by H3K27me3, a repressive mark enriched on the inactive X-chromosome, but not by active histone marks H3K4me3 nor H3K27ac that are depleted on the inactive X (Fig. 1d). Interestingly, from *Xist’s* PIRCh-seq signal, it is possible to infer which domain of this lncRNA is associated with chromatin. Within the *Xist* locus, the 5′ domain of *Xist* displays significantly more substantial enrichment in H3K27me3 PIRCh-seq as compared to other regions along the RNA (highlighted by the gray box, Fig. 1d), consistent with previous findings that this is the domain potentially associated with chromatin (repC domain) [30–32]. Conversely, coding genes such as *Actb* and *Eef2* were not enriched on chromatin with the same set of modifications (Additional file 1: Figure S2E-F). These results were obtained from three different cell lines in two species and indicate that PIRCh-seq is able to identify histone modification-specific chromatin-associated lncRNAs transcriptome-wide.

PIRCh-seq can also be utilized to identify novel histone modification-specific chromatin-enriched lncRNAs. In our NPC PIRCh-seq, a lncRNA upstream of the *Nr2f1* gene, *lnc-Nr2f1*, was retrieved by the promoter marks histone H3K4me3 (*P* < 0.05), but not enhancer-associated nor repressive modifications (H3K27ac and H3K27me3), indicating that this lncRNA may preferentially associate with H3K4me3 regions (Fig. 1e). Recently, *lnc-Nr2f1* was reported to play a critical role in regulating neurodevelopmental disorders [33]. In order to further validate the chromatin-RNA association of this lncRNA, we retrieved *lnc-Nr2f1* RNA and mapped its associated DNA elements in NPCs (ChIRP-seq experiment). Overlaying *lnc-Nr2f1* ChIRP-seq with ChIP-seq data of the histone modifications confirmed that *lnc-Nr2f1* does bind to genomic locations with H3K4me3 (Fig. 1f, g), which further confirms that the PIRCh approach can retrieve lncRNAs specifically associated with certain modifications. In addition, gene ontology analysis of *lnc-Nr2f1* ChIRP-seq peaks using GREAT [34] suggests that *lnc-Nr2f1* regulates cerebellar cortex development (Additional file 1: Figure S2G, *P* < 10^−5^), consistent with previous findings regarding the function of this lncRNA. These results not only demonstrate the reliability of PIRCh-seq in identifying chromatin-associated ncRNAs, but also suggest potential application of the histone modification-specific PIRCh-seq approach in predicting their functions.

### PIRCh-seq enriches lncRNAs on chromatin with low nascent transcription

**Fig. 2:**
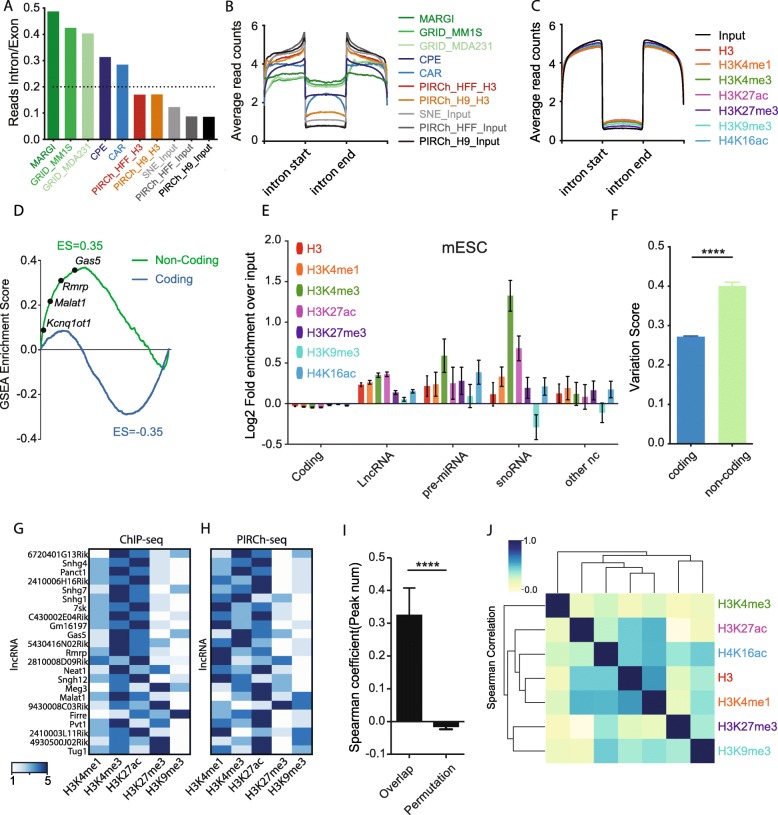
ncRNAs are enriched on chromatin compared with protein coding transcripts. **a** Ratio of intronic over exonic reads obtained from different chromatin-RNA association sequencing technologies (MARGI, GRID, CPE, CAR, and PIRCh) versus input controls in multiple cell lines. **b** Normalized average read coverage around introns from different chromatin-RNA association sequencing technologies (MARGI, GRID, CPE, CAR, and PIRCh) versus input controls in multiple cell lines. **c** Normalized average read coverage around introns from histone modification-specific PIRCh-seq profiles (colored) and inputs (black) in mouse embryonic stem cells (mESCs). **d** Gene set enrichment analysis (GSEA) shows highly statistical enriched (FDR = 0, *P* < 0.0001) non-coding genes (green) and depleted coding genes (blue) on histone H3 in mESCs. Genes were ranked by their histone H3 PIRCh enrichment scores. **e** Average fold enrichment (calculated by limma in R) of the coding gene, lncRNA, pre-miRNA, snoRNA, and other ncRNAs from histone modification-specific PIRCh-seq profiles (namely H3, H3K4me1, H3K4me3, H3K27ac, H3K27me3, H3K9me3, and H4K16ac) in mESC. Error bar shows the standard deviation from the mean. **f** Average variation score of the PIRCh-seq signals for the coding versus non-coding genes (****P < 0.0001, two-tailed Welch’s *T* test). Error bar shows the standard deviation from the mean. **g** Heatmap displaying the ranking of the ChIP-seq enrichment of the chromatin binding sites of 23 lncRNA. The 23 lncRNAs are chromatin enriched from PIRCh-seq, and the chromatin binding sites are obtained from ChIRP/CHART/RAP/GRID-seq profiles from the LnChrom database. Colors represent ranking from 1 to 5. **h** Heatmap shows the ranking of PIRCh-seq enrichment of the same lncRNAs in **g**. Colors represent ranking from 1 to 5. **i** Bar plot of the Spearman correlation coefficients between the ranking in **g** and **h** for each lncRNA versus random permutation (****P < 0.0001, two-tailed Welch’s *T* test). **j** Unsupervised clustering of the Pearson correlation coefficients matrix of the histone modification-specific PIRCh-seq profiles based on the enrichment scores from the 258 chromatin-associated ncRNAs in mESC

Various techniques have been developed to study ncRNA functions on chromatin. For instance, ChIRP [25], CHART [35], and RAP [36] are RNA-centric methods that profile DNA binding sites genome-wide of one target RNA at a time. Many investigators have isolated chromatin-associated RNAs from stringent nuclear or chromatin fractionation [17–19]. In addition, recent methods such as GRID-seq and MARGI can be applied in mapping the global RNA-chromatin interactome [20, 21]. Comparatively, chromatin fractionation and sequencing detects chromatin-associated RNA without delineating the specific chromatin states that specific RNAs prefer. Furthermore, proximity ligation methods predominantly detect nascent RNAs co-transcriptionally tethered to chromatin by RNA polymerase, confounding background signal from all RNAs in the process of transcription. Thus, to evaluate the level of nascent transcription from PIRCh, we compared our PIRCh-seq results in H9 and HFF with that from GRID-seq [20], MARGI [21], che-RNA isolation (named CPE “chromatin pellet extract” for experiment and SNE “soluble-nuclear extract” for background control) [19], and chromatin-associated RNAs (CAR) [17]. These experiments were all performed in human cell lines. We found that the ratios of intronic reads in PIRCh-seq profiles were significantly lower than those from previously reported methods (*P* < 0.01, *T* test) and were almost comparable with input RNAseq from bulk cultured cells (Fig. 2a). Moreover, by averaging signals over the entire transcriptome centered by introns from all the existing methods (the “Methods” section), we found PIRCh was more effective in obtaining mature RNAs than extant chromatin-RNA enrichment methods, based on the higher signal over exons than introns (Fig. 2b). We obtained similar findings in other cell types and with every tested histone modification (Fig. 2c, PIRCh-seq of V6.5 mouse ES cells with histone H3 and 6 histone modifications). These results demonstrate that PIRCh-seq consistently generates a significantly lower level of intronic reads with multiple histone modifications than existing methods, and therefore is able to preserve regulatory interactions *in trans* between lncRNAs and chromatin.

To further estimate the level of nascent transcription, we then integrated each histone modification-specific PIRCh-seq profile with its corresponding ChIP-seq dataset in the same cell line (V6.5 mESCs) [37], and asked whether the PIRCh-seq signal of each RNA correlated with the nearby ChIP-seq signal carrying the corresponding modification (see the “Methods” section). Our results suggest that there was no significant correlation with these two sets of signals (Additional file 1: Figure S3A-E), confirming that the recovery of nascent transcripts from PIRCh-seq is negligible. One likely explanation is that glutaraldehyde is a longer span crosslinking reagent (5 carbons instead of 1 carbon in formaldehyde) that is capable of capturing more regulatory RNAs which are able to interact over a longer range compared to nascent transcripts [38, 39]. These results suggest that the majority of PIRCh-seq-enriched chromatin-associated RNAs are mature RNAs with introns spliced out, which allows PIRCh-seq to identify more chromatin-associated RNAs with low abundance, such as many ncRNAs.

### PIRCh-seq identifies ncRNAs associated with specific histone modifications

Because PIRCh-seq enables transcriptome-wide annotation of chromatin-RNA association, we next determined whether various types of RNA (especially coding RNAs versus ncRNAs) are differentially affiliated with chromatin. We first filtered the expressed RNAs in edgeR [40] and normalized the RNA read counts in limma [41] for both the PIRCh-seq and input samples (the “Methods” section, Additional file 1: Figure S4A-B). We then defined a PIRCh enrichment score by dividing the normalized read counts in PIRCh over input, and ranked all the transcripts by their enrichment scores in H3 PIRCh-seq. To test whether ncRNAs were enriched on chromatin, we performed a gene set enrichment analysis (GSEA) [42] of the annotated coding and non-coding RNAs. We found that ncRNAs, but not coding RNAs, were indeed highly enriched on chromatin and many known ncRNAs were top ranked in terms of chromatin enrichment scores (Fig. 2d). Next, we performed PIRCh-seq with antibodies specific to distinct histone modifications in mESCs. Similar to the enrichment on histone H3, we expect that ncRNAs should be highly ranked by the average fold enrichment of the histone modification-specific PIRCh-seq signal versus the corresponding input, among all the expressed genes. Indeed, compared with mRNAs, we found that in most cases (22 out of 28), the average enrichment scores of the annotated lncRNAs, pre-miRNAs, and snoRNAs, as well as other ncRNAs, were significantly higher on H3 and multiple histone modified chromatin than coding genes (Fig. 2e, *P* < 0.05, *T* test). We then checked the distributions of the expressed and chromatin-associated RNAs on histone H3 and chromatin with other modifications in mESCs, and found that ncRNAs were significantly more frequent on chromatin compared with mRNAs (Additional file 1: Figure S4C), serving as additional evidence that ncRNAs are more enriched on chromatin in general. Furthermore, when we defined a variation score which measured the standard deviation of the chromatin association enrichment scores across each histone modification for every expressed RNA, we concluded that ncRNAs are significantly more variable than mRNAs (Fig. 2f, *P* < 0.001, *T* test). This suggests that non-coding transcripts are more differentially enriched at distinct chromatin states, consistent with the potential regulatory function divergence of lncRNAs, and naturally prioritizes downstream studies of lncRNAs by activity.

We then sought to characterize the ncRNAs significantly on chromatin in mESC from their PIRCh-seq profiles. We considered PIRCh-seq biological replicates versus the inputs in limma [41] and defined an RNA with chromatin association by *P* value < 0.05 (the “Methods” section). Using this cutoff, we identified 258 chromatin-associated ncRNAs in mESC which were enriched in at least 1 of the 6 histone modification-specific PIRCh-seq profiles (Additional file 2: Table S1). To further evaluate the performance of the PIRCh approach, we compared our PIRCh-seq-enriched lncRNA results with 96 published RNA-chromatin association profiles from ChIRP/CHART/RAP/GRID-seq datasets, collected by LnChrom [43]. We found a total of 23 lncRNAs, including *Xist*, *Firre*, *Rmrp*, and *Tug1*, were also expressed in our mESCs. All 23 lncRNAs were positively enriched in PIRCh, and 14 were significant with *P* < 0.05, reaffirming the sensitivity of the PIRCh approach in identifying chromatin-associated lncRNAs. Furthermore, we wanted to validate whether the PIRCh lncRNA enrichment patterns were consistent with results obtained from published orthogonal methods. We hypothesized that if a lncRNA is able to associate with DNA elements marked by a specific histone modification, its genomic binding sites from ChIRP/CHART/RAP/GRID-seq experiments should greatly overlap with corresponding ChIP-seq peaks associated with the same modification. We then obtained the genomic binding sites (peaks) of the 23 lncRNAs from the aforementioned experiments, and found the ratio of this overlap from published data (Fig. 2g) is highly correlated with the corresponding PIRCh-seq signal among most of the lncRNAs (Fig. 2h). The Spearman correlation coefficients of the ratio of the overlap ChIP-seq peaks [37] with the lncRNA’s PIRCh-seq enrichment scores in the same cell line were significantly higher than random permutations (Fig. 2i, *P* < 0.0001). These results further confirm that PIRCh-seq reliably identifies chromatin-associated lncRNAs.

Conversely, we hypothesized that certain ncRNAs are enriched at chromatin with distinct types of DNA regulatory elements, and asked whether gene regulatory elements could be naturally differentiated via chromatin-ncRNA association. We then calculated the pairwise Pearson correlation of all chromatin states based on the PIRCh-seq enrichment scores of 258 chromatin-associated ncRNAs. It is clear that the enhancer-like states (H3K27ac, H4K16ac, H3K4me1) were clustered together, then the repressive histone modifications (H3K27me3, H3K9me3), while the promoter (H3K4me3) was grouped in a distinct cluster (Fig. 2j). Interestingly, the PIRCh-seq signal of histone H3 clustered closest with H3K4me1 (Pearson’s correlation *r* = 0.89). We observed that H3K4me1 ChIP-seq signal from the same cells as above covers three to four times the genomic regions than other chromatin modifications, which may reflect the differential sensitivities of the different antibodies for ChIP (Additional file 1: Figure S4D).

### PIRCh-seq classifies functional ncRNAs via chromatin association

**Fig. 3:**
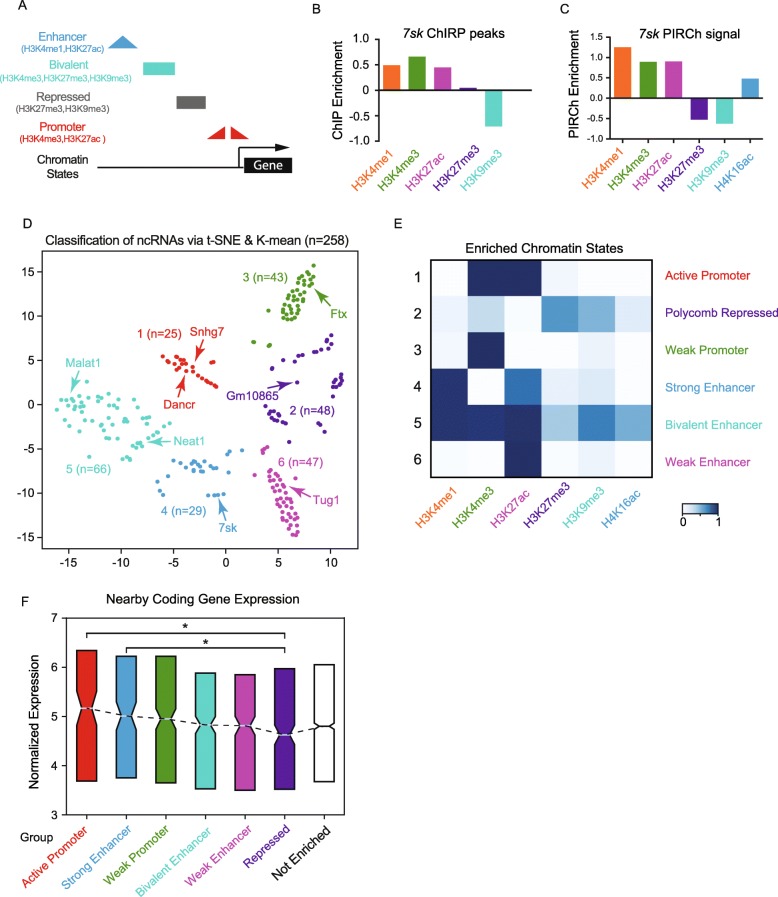
PIRCh-seq classifies functional ncRNAs via chromatin state association. **a** Summary of histone modifications representing distinct regulatory patterns. **b** The enrichment of the *7sk* ChIRP-seq peaks overlap with different histone modification ChIP-seq peaks in the same cell line (mESC). A positive value indicates the ChIRP-seq peaks are highly enriched with ChIP-seq peaks compared to random, and a negative value indicates depletion. **c** The PIRCh enrichment score of the lncRNA *7sk* in mESC from distinct histone modification-specific PIRCh-seq experiments. A positive value means enriched, and a negative value means depleted. **d** Classification of the PIRCh-seq identified chromatin-associated ncRNAs (*n* = 258) in mESC. Scatter plot shows the t-SNE result on PIRCh-seq enrichment score matrix and annotated by *K*-means clustering. **e** Functional classification of histone-specific chromatin-RNA association patterns defined by chromHMM algorithm. **f** Box plot of the expression of the coding genes nearby (± 100 Kb) each group of PIRCh-clustered ncRNAs defined in **d**. Center lines represent mean values; box limits represent the interquartile range. The expression of the coding genes that are close to the ncRNAs in the “repressed” group is significantly lower than those in the “active promoter/enhancer” group (*P* < 0.05, two-tailed Welch’s *T* test). Genes close to un-enriched ncRNAs are shown as controls

Different gene regulatory elements—such as enhancers, promoters, insulators, and silenced elements—carry distinctive and characteristic histone and DNA modifications (Fig. 3a) [44]. We noticed that 14–25 ncRNAs in HFF and H9 respectively were also reported as “essential” ncRNAs with functions through CRISPRi screening [6]. We then hypothesized that specific modification-enriched ncRNAs regulate each of these elements, and thereby, the functions of ncRNAs can be classified by their divergent chromatin modification enrichment. Hence, PIRCh-seq is anticipated to classify and associate ncRNAs with functions such as promoter, enhancer, silencer, or insulator. To test this hypothesis, we analyzed *7sk*, a well-known regulator of RNA polymerase II elongation that resides at enhancers, promoters, and super-enhancers [45], consistent with its role in enhancer-promoter interactions. From *7sk* ChIRP-seq data in mESC, we noticed that its chromatin occupancy sites greatly overlapped with ChIP-seq peaks of H3K4me1, H3K4me3, and H3K27ac in the same cell type (Fig. 3b), confirming an active function of *7sk*. Consistently, PIRCh-seq signal of *7sk* in mESC was also enriched at chromatin carrying these three histone modifications, but depleted of repressive modifications such as H3K27me3 and H3K9me3 (Fig. 3c), suggesting the possibility to extrapolate lncRNA function using PIRCh-seq.

We then analyzed all 258 PIRCh-enriched ncRNAs and sought to categorize their functions based on their PIRCh-seq signals. We found that these ncRNAs associate with chromatin in a combinatorial pattern, similar to those observed in ChIP-seq performed on histone modifications (Additional file 1: Figure S5A). H3K27ac, H3K4me3, and H3K4me1 were the top three most favored chromatin states that interacted with ncRNAs, consisting of 88% of the enriched ncRNAs in mESC. As we know from histone ChIP-seq, instead of each individual modification, a combinatorial pattern of multiple modifications better classifies the functions of DNA elements [46]. A machine learning strategy employing hidden Markov model, named chromHMM, which automatically learns the major combinatorial patterns, was applied successfully to classify DNA elements based on histone modifications [47, 48]. We then inquired if a similar strategy could be used to classify chromatin-associated ncRNAs and examine if the functions of these ncRNAs could be distinguished based on their association with histone modifications. To investigate this relationship transcriptome-wide, we started from a 258 by 6 matrix of enrichment scores in mESC, where each row was an enriched ncRNA as defined above, each column was a histone modification, and each element of the matrix represented the enrichment score of the corresponding ncRNA on the specific modified chromatin (the “Methods” section). We then applied *K*-means clustering on the matrix, where the number of *K*’s was determined by the Silouette method [49]. This analysis yielded six distinct groups of chromatin-associated ncRNAs, which were visualized in a two-dimensional projection of t-distributed stochastic neighbor embedding (tSNE) (Fig. 3d). Within these 258 chromatin-associated ncRNAs, 247 are lncRNAs, and many well-studied ncRNAs, such as *7sk* [45], *Neat* [10] and *Malat1* [50], and *Dancr* [51], naturally clustered into groups with distinct function. Interestingly, 14 lncRNAs were also reported to have a biological function based on LncRNAdb [52], and 8 out of 56 were predicted bivalent in mESCs (Additional file 1: Figure S5B, odds ratio = 4.4, *P* < 0.01, chi-square test). In addition, for each cluster, we evaluated the relative contributions of each histone modification based on the enrichment pattern of the chromatin-associated RNAs, and defined the clustered states by active promoters, heterochromatin, weak promoter, strong enhancer, bivalent, and weak enhancer (Fig. 3e). Overall, we partially recapitulated the chromatin classifications by applying the chromHMM algorithm based on PIRCh-seq profiles with six well-studied histone modifications [47]. These results suggest that the chromatin association of ncRNAs can be used to classify ncRNAs that might have functional implications.

Although tens of thousands of non-coding transcripts were discovered in the past few years, only a small portion that function *in cis* or *trans* through chromatin organization were consolidated [53, 54]. Since the PIRCh approach cannot pinpoint the exact binding sites of chromatin-associated lncRNAs, it does not directly predict whether each lncRNA is functioning *in cis* or *trans*. Instead, PIRCh provides more information about the epigenetic function of the lncRNA, in context of the histone modifications it associates with. Our analysis suggests that chromatin-associated lncRNAs function both *in cis* and *trans*. For example, when we calculated the nearby (± 100 Kb) coding gene expression of the PIRCh-clustered ncRNAs in Fig. 3d, we observed that lncRNAs were monotonically decreasing from the more active to more repressive groups; additionally, the nearby coding gene expression of the “Active Promoter” and “Strong Enhancer” lncRNA groups were significantly higher than that of the group “Repressed” ncRNAs (Fig. 3f, *P* < 0.05, *T* test). However, when the chromatin-associated ncRNAs were grouped based on their enrichment with each histone modification, no significant expressional differences were observed from nearby coding genes (Additional file 1: Figure S5C), e.g., compared H3K27me3 vs H3K27ac. No similar trends were observed in the expression patterns of the ncRNAs themselves (Additional file 1: Figure S5D). Nevertheless, not all the lncRNAs were enriched in our PIRCh experiment function *in cis*. When we integrated each histone modification-specific PIRCh-seq profile with its corresponding ChIP-seq signal at the genomic loci of the chromatin-enriched ncRNAs, no statistical correlation was observed (Additional file 1: Figure S3), suggesting that some lncRNAs can function *in trans*. However, further investigations using ChIRP and ChIP analysis preferably at single cell level are still required to fully uncover their regulatory patterns on chromatin.

### Cell type-specific chromatin association of ncRNAs

**Fig. 4:**
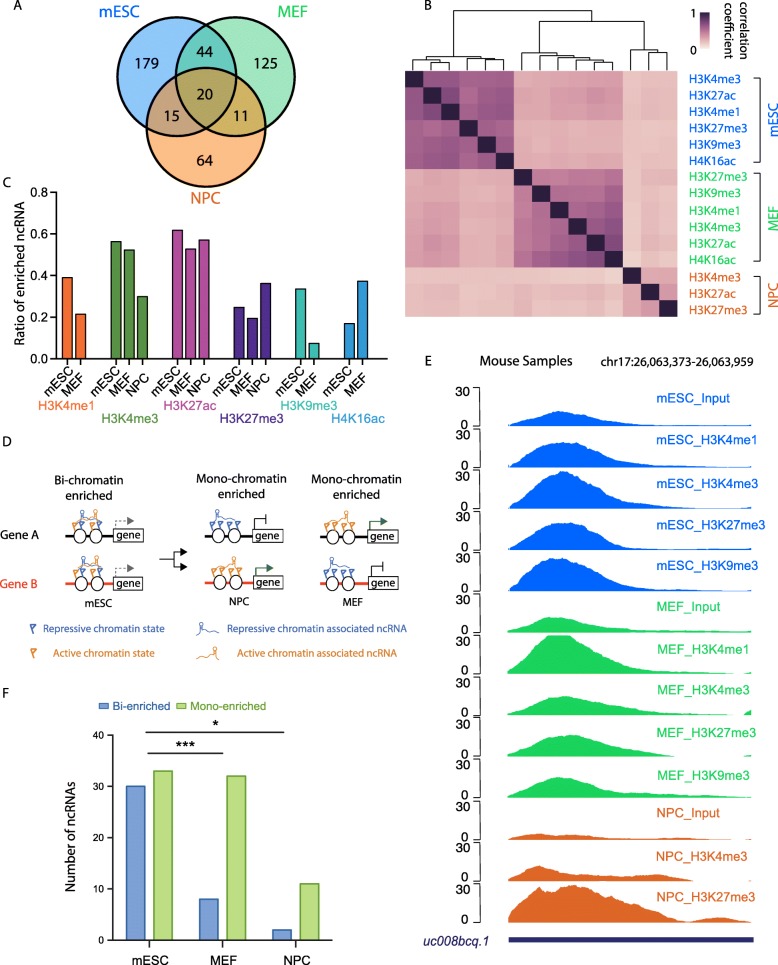
Cell type-specific chromatin association of ncRNAs. **a** Number of chromatin-enriched RNAs in mESC, MEF, and NPC. **b** Unsupervised clustering of the Pearson correlation coefficient matrix of the histone modification-specific PIRCh-seq profiles in mESC, MEF, and NPC, based on the enrichment scores from the 458 chromatin-associated ncRNAs in each cell type. **c** Ratio of the chromatin-enriched ncRNA under each chemical modification over the total number of enriched ncRNAs in mESC, MEF, and NPC. **d** Schematic illustration of how RNAs enriched on both the repressive and active chromatin (bi-chromatin enriched) and either the repressive or active chromatin (mono-chromatin enriched). **e** UCSC tracks of the normalized PIRCh-seq signal at the lncRNA *uc008bcq.1* locus in mESC, MEF, and NPC. *uc008bcq.1* is bi-chromatin enriched in mESC, but mono-chromatin enriched in MEF and NPC. **f** Number of ncRNAs that are bi-chromatin enriched or mono-chromatin enriched in mESC, MEF, and NPC (***P < 0.001, *P < 0.05, chi-square test)

It is known that ncRNAs are differentially expressed in distinct cell types and perform specific cellular functions. Therefore, we sought to check whether the patterns of ncRNA-chromatin association diverge in distinct mouse cell types, and how these patterns contribute to their cell type-specific functions. We then performed PIRCh-seq on MEF cells and analyzed the profiles in an identical fashion to the mESC data. Similar to the mESC results, we observed that PIRCh-seq identified lncRNAs enriched on chromatin with low nascent transcription (Additional file 1: Figure S6A), and non-coding transcripts were consistently more enriched on chromatin compared with protein coding gene in MEF cells (Additional file 1: Figure S6B-C), validating these conclusions in distinct cell types. We then performed a similar enrichment analysis on MEF and NPC PIRCh-seq profiles and obtained 200 and 110 chromatin-associated ncRNAs, respectively (*P* < 0.05). The chromatin-enriched ncRNAs in MEF also form a combinational pattern with multiple histone modifications (Additional file 1: Figure S6D-E). As a negative control, the IgG PIRCh was tested in tandem with the other chromatin modification PIRCh experiments performed in MEF, take *PVT1* as an example (Additional file 1: Figure S6F). Differential analysis of PIRCh groups over IgG control revealed that only 1 out of 200 PIRCh-enriched ncRNA over input was also enriched in IgG, evincing the high specificity of our method in identifying the chromatin-associated ncRNAs. In our analysis, a total of 458 chromatin-enriched ncRNAs were identified in 3 cell types, 20 of which were enriched in all 3 cell types (Fig. 4a). We then calculated the Pearson correlation coefficient matrix based on the enrichment scores of these 458 ncRNAs, where the expression divergence of the same lncRNA in different cells was normalized. Unsupervised clustering of this correlation matrix suggested that the cell type specificity was the dominant factor which determines ncRNA-chromatin association (Fig. 4b).

Embryonic stem cells are characterized by their pluripotency—the ability to give rise to multiple cell types. The chromatin state in ES cells is reported to be more flexible than those of differentiated cells [55]. Interestingly, compared with those of the more differentiated cells (MEF and NPC), the ncRNA-chromatin association in mESCs showed a higher correlation coefficient among distinct histone modifications, suggesting less specificity in chromatin-ncRNA association in mESCs than those in differentiated cells (Fig. 4b). In addition, we analyzed the percentage of enriched ncRNA versus total expressed ncRNA in each cell type for every tested chromatin modification, and found significantly more ncRNAs enriched on chromatin with H3K9me3 in ES cells when compared with MEF (Fig. 4c, *P* < 0.05, chi-square test), but fewer on chromatin with H4K16ac. This result may reflect the joint presence of activating and repressive histone marks on genome regions, termed bivalent [56] and trivalent chromatin domains [57] in ES cells. We identified ncRNAs which were associated with both active and repressive histone marks consistent with bivalency, while others associated with strictly active or repressive marks (Fig. 4d). Since PIRCh-seq enabled us to identify cell type- and histone modification-specific ncRNA-chromatin associations, we first screened for ncRNAs which were enriched at both active and repressive chromatin in ES cells but only enriched in either active or repressive markers in differentiated cells. We found several ncRNAs of this description. For example, ncRNA *uc008bcq.1* is broadly enriched in ES cells with high PIRCh-seq signals associated H3K4me1, H3K4me3, H3K27me3, and H3K9me3 modifications, but enriched only on active chromatin of H3K4me1 in MEF and repressive chromatin of H3K27me3 in NPC, implying lineage-specific resolution of chromatin associations (Fig. 4e). Interestingly, there were dozens of such ncRNAs that are distinctly enriched in certain cell types. Since ES cells possess a higher potential to differentiate into multiple lineages, and hence more poised chromatin states, we expected more bivalent-enriched (“bi-enriched” for short) and fewer mono-enriched ncRNAs in mESC compared with more differentiated cells such as MEF and NPC. In mESC, we found 30 bi-enriched and 33 mono-enriched ncRNAs, while in MEF, we found only 8 bi-enriched but 32 mono-enriched ncRNAs; lastly, in NPC, we found 2 bi-enriched and 11 mono-enriched ncRNAs (Fig. 4f, *P* < 0.01 for MEF and *P* < 0.05 for NPC, chi-square test). As well known, ES cells are enriched for bivalent chromatin, comprised of both H3K4me3 and H3K4me27 on the same nucleosomes [58, 59] that mark “poised” developmental genes that may either turn on (marked by H3K4me3 only) or turn off (marked by H3K27me3 only) in distinct differentiated cell types. We interpret our PIRCh-seq result to mean that certain ncRNAs can associate with H3K27me3 or H3K4me3 in either the bivalent or the monovalent state, and we are observing the combinatorial action of the histone code in cell differentiation. These results indicate that ncRNAs may play distinct functional roles by either enhancing or repressing gene expression or both in certain cell types, conducted by affixing to either active or repressive chromatin or both.

### Single-stranded RNA regions as candidate mediators of chromatin association

**Fig. 5:**
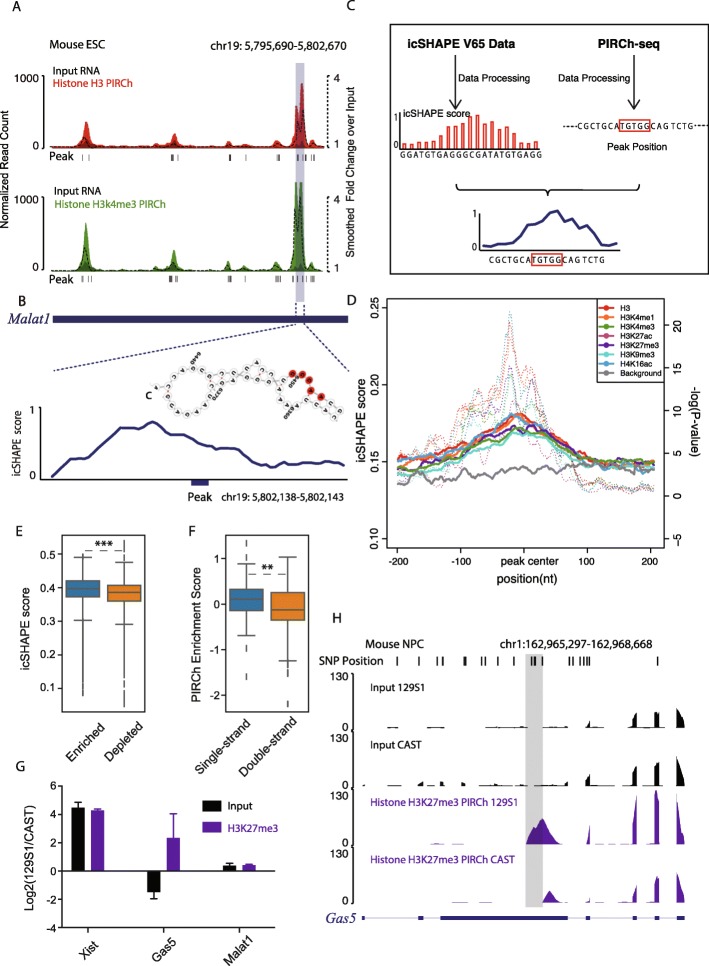
RNAs with single strand are more likely to associate with chromatin. **a** UCSC track of the normalized input (black) and H3 (red) and H3K4me3 (green) PIRCh-seq signals of lncRNA *Malat1* in mESC. Dash lines represent fold change of PIRCh-seq over input and smoothed by 500 bp sliding windows. The boxed region represents the most enriched domain on *Malat1*. Bottom peaks are chromatin-enriched sites on *Malat1*. **b** Structure profile from icSHAPE and structural prediction from RNAfold around a zoom in chromatin-associated peak on lncRNA *Malat1*. **c** Computational workflow to integrate RNA secondary structure information from icSHAPE and chromatin enrichment information from PIRCh-seq to study the structural preference of chromatin-RNA association. **d** Average diagram of icSHAPE scores around all PIRCh-seq peaks under different histone modifications (colored solid line) versus a randomly selected background (gray solid line). *P* values (colored dash line) were estimated by using two-tailed Welch’s *T* test on every position between PIRCh-seq profiles over background. **e** Box plot of the icSHAPE score of PIRCh-seq enriched vs depleted RNAs (***P < 0.001, two-tailed Welch’s *T* test). Center lines represent mean values; box limits represent the interquartile range; whiskers each extend 1.5 times the interquartile range; dots represent outliers. **f** Box plot of the PIRCh-seq enrichment scores of the top 100 most single-stranded RNAs versus the top 100 most double-stranded RNAs based on icSHAPE scores (**P < 0.01 two-tailed Welch’s *T* test). Center lines represent mean values; box limits represent the interquartile range; whiskers each extend 1.5 times the interquartile range; dots represent outliers. **g** Relative allele-specific RNA expression and chromatin enrichment of lncRNAs *Xist*, *Gas5*, and *Malat1* in the 129S1 allele versus the CAST allele of NPC. The *Y*-axis represents the log2 fold change of the allelic signals in 129S1 over CAST. The 129S1 version of lncRNA *Xist* is highly expressed and also enriched at chromatin with H3K27me3 modification. Both alleles of lncRNA *Malat1* were almost equally expressed and enriched. The 129S1 version of *Gas5* was lowly expressed but highly enriched on chromatin compared to the CAST version of the same gene. **h** Normalized allele-specific input and histone H3K27me3 PIRCh-seq signals in the 129S1 and CAST alleles. Top shows single nucleotide polymorphisms (SNP) positions that distinguish the alleles

As a key player in the central dogma of biological regulation, RNA and its ability to adopt specific structures is intimately involved in every step of gene expression. Previously, multiple approaches have been described in order to probe RNA secondary structure transcriptome-wide in vitro [60] and in vivo [32, 61] in mammalian cells, revealing structural principles of RNA-protein interactions. Correspondingly, we noted that RNA enrichment on chromatin occurs in a domain-specific manner based on our PIRCh-seq data. For instance, the repC domain of *Xist* is dramatically more enriched on chromatin carrying H3K27me3 modifications (highlighted by the gray box, Fig. 1d). *Malat1* is another well-studied chromatin-associated lncRNA which binds to active chromatin [10]. Instead of attaching to chromatin across the entire transcript, we noticed from the H3K4me3 PIRCh-seq signal that there were certain regions on *Malat1* which were more closely associated with chromatin than the rest bases on the transcript (Fig. 5a). Interestingly, these regions tend to be single stranded according to both 2′ hydroxyl acylation profiling experiments (icSHAPE data) and RNA secondary structure predictions from RNAfold [62] (Fig. 5b). This led us to investigate whether there are structural preferences involved in RNA-chromatin association (Fig. 5c). We first obtained a transcriptome-wide and per-base RNA secondary structure profile from icSHAPE data measured in mESCs [61]*.* A high icSHAPE score suggests a greater probability that a base is single stranded. We then applied a 5-base sliding window method to identify the enriched sites (peaks) on each RNA which interacted with chromatin from PIRCh-seq, compared with our input control (see the “Methods” section). We then overlaid the structural profiles from icSHAPE on top of all the histone modification-specific PIRCh-seq peaks centered by the peak summits and generated an average structural profile for each modification. Our results show that bases ~ 5–10 nt upstream of the chromatin-associated peaks are more likely to be single stranded (Fig. 5d). To test the significance of this single-strand preference, we performed two-tailed Welch’s *T* tests by comparing all the icSHAPE scores of the bases from PIRCh-seq peaks with those from a randomly selected background, and found this phenomenon was significant with *P* < 10^−5^. We then asked whether RNAs containing a greater number of single-stranded bases are more likely to be associated with chromatin. We separated expressed RNAs into two groups based on chromatin enrichment or depletion, and calculated the average icSHAPE scores for every RNA in each group. We noticed that, on average, RNAs enriched on chromatin tended to be more single stranded with higher icSHAPE scores (Fig. 5e, *P* < 0.001, *T* test). Similarly, we took the top 100 most single-stranded RNAs and top 100 most double-stranded RNAs based on their average icSHAPE scores and confirmed that the average chromatin enrichment scores of the most single-stranded RNAs were significantly higher than those of the double-stranded RNAs (Fig. 5f, *P* < 0.01, *T* test). These results suggest that RNAs containing more single-stranded regions are more likely to associate with chromatin.

### Single nucleotide variants and RNA modifications that alter chromatin association

Genetic variation can alter RNA structure and function in vivo. Single nucleotide polymorphisms (SNPs) comprise the most prevalent source of variation, and SNPs that alter RNA secondary structures, termed “riboSnitches” (a fusion of SNP and riboswitch), are a recently appreciated source of non-coding variants associated with human diseases [63]. We therefore asked whether different alleles of the same RNA may differentially associate with chromatin, and if so, how is it related to the RNA structure? In order to answer those questions, we performed PIRCh-seq in the NPC line that is derived from the F1 hybrid offspring of two mouse parental lines (129S1 and CAST) with a high density of SNPs across the genome (~ 1 SNP per 100 nucleotides). We first built the reference genomes for each mouse line and aligned the raw reads to 129S1 and CAST separately with 0 mismatches to reduce false-positive hits. Reads mapped to either 129S1 or CAST were counted to construct the allele-specific RNA expressions and chromatin enrichment profiles (see the “Methods” section). First, we looked at whether allelic RNA chromatin association is related to allelic expression. From allele-specific PIRCh-seq analysis, we found that for most RNAs, allelic expression from the two alleles determines allelic chromatin association pattern. For example, it is known that only the 129S1 version of the lncRNA *Xist* is expressed in this cell line. Consistent with allelic expression, we found that enrichment of *Xist* in H3K27me3 modification is much higher in 129S1 versus the CAST version of the lncRNA (Fig. 5g). An additional example is the lncRNA *Malat1* in which both the 129S1 and CAST alleles are almost equally expressed. As predicted, we observed unbiased enrichment on chromatin for both alleles. Moreover, we discovered several lncRNAs that are enriched on chromatin in an allele-specific manner independent of the expression levels from the two alleles (Additional file 3: Table S2). For example, *Gas5* is a lncRNA that binds to PRC2 complex and mediates transcriptional repression [64]. We found that *Gas5* is enriched in H3K27me3 modification, consistent with its understood repressive function. Notably, even though the CAST version of *Gas5* was threefold more expressed in the input sample, the 129S1 allele was fourfold more enriched on chromatin carrying H3K27me3 modification (Fig. 5g, *P* < 0.05, *T* test), suggesting that 129S1 allele of *Gas5* preferentially associates with chromatin. To further investigate the mechanism under *Gas5* allele-specific enrichments, we predicted the secondary structure of the 129S1 and CAST version of *Gas5* using RNAfold (Additional file 1: Figure S7A, B), and found that several riboSnitches (1057 A/T, 1059 G/T, 1134 C/T, CAST (mm9)/129S1) converted one of the chromatin binding sites of *Gas5* from single stranded in 129S1 to double stranded in CAST and thus depleted its association with repressive chromatin in the latter (Fig. 5h). Consistent with this prediction, when we calculated the icSHAPE score obtained from mESC containing 129S1 allele [65] for the *Gas5* allelic-enriched region in H3K27me3, we concluded that the region is more likely to be single stranded (Additional file 1: Figure S7C).

Another major factor that can influence RNA structure is RNA modification, such as the *N*6-methyladenosine (m^6^A) modification. Previous studies have shown that m^6^A can alter base-pairing thermodynamics and destabilize RNA duplexes [61, 66, 67]*.* We also evaluated whether RNA modifications affect RNA-chromatin association. We integrated PIRCh-seq data with the transcriptome-wide profiles of RNA m^6^A modifications in mESCs from our previous study [68], and found the distribution of PIRCh-seq peaks along the transcripts is similar to that of m^6^A modified regions (Additional file 1: Figure S8A). When we overlaid m^6^A signals on top of PIRCh-seq peaks, we found that RNA bases associated with chromatin are generally more m^6^A modified (*P* < 10^−5^ in H3, Additional file 1: Figure S8B-C). These results may reflect the tendency of m^6^A to induce RNA single-stranded regions that coincide with elements for chromatin association, or due to additional mechanisms that jointly impact chromatin association and RNA modification.

## Discussion

### PIRCh-seq identifies chromatin-associated RNAs genome-wide

A large and growing body of literature has investigated protein-RNA interactions. The development of approaches such as RIP [13], CLIP [14], fRIP [15], and CARIP [16] has enabled the successful elucidation of many RNAs associated with proteins, including multiple chromatin regulators. Studies have also shown that many lncRNAs function through DNA/chromatin interaction. Previously described techniques such as ChIRP-seq and CHART-seq have been used to identify genome-wide binding sites of specific lncRNA to chromatin. However, these methods require prior knowledge of which particular lncRNAs are capable of binding to chromatin before ChIRP-seq or CHART-seq can be applied. Furthermore, ChIRP or CHART is limited to examining one chromatin-associated RNA at a time. In this study, we describe a new technology, PIRCh-seq, which enables a global profiling of chromatin-associated RNAs through a robust method to crosslink endogenous RNA-chromatin interactions in living cells. Compared with current methods which predominantly detect nascent RNAs co-transcriptionally tethered to chromatin by RNA polymerase, PIRCh-seq significantly reduces the influence of nascent transcripts and more clearly reveals relationships between chromatin and its associated ncRNAs. Although the PIRCh approach cannot pinpoint the exact binding sites of the chromatin-associated lncRNAs, and therefore does not inform whether each lncRNA is functioning *in cis* or *trans*, it is able to provide a significantly higher ratio of mature RNAs. Examples of some well-studied cases, such as *Xist*, *7sk*, *H19*, and *KCNQ1OT1*, demonstrate that PIRCh-seq is likely generalizable to the majority of ncRNA. Additionally, PIRCh-seq identifies novel chromatin-associated lncRNAs and not only provides potential targets for mechanistic studies using ChIRP and CHART, but could also be extended to reveal the function and mechanisms of lncRNAs which are disease-relevant. However, the PIRCh-seq approach, like RIP/CLiP-seq-like methods, may also be heavily contaminated with co-purified mRNA species that often compose more than 50% of RNA material. Therefore, further experimental and analytical improvements are required to truly capture chromatin-associated ncRNAs.

### PIRCh-seq classifies ncRNA putative function via histone modification and cell type-specific chromatin-RNA association

Another major advantage of the PIRCh method is that it utilizes antibodies to pull down chromatin with specific chemical modifications and thereby enables the classification of chromatin-associated ncRNAs with putative functions such as promoter, enhancer, silencer, or bivalent. Since we performed PIRCh-seq with various histone modification antibodies and in different human and murine cell types, the dataset provides rich resources to study chromatin-associated ncRNAs in mammalian cells. In addition, different cell types and histone modifications did not show much technical variation, confirming that PIRCh-seq may be a useful technology to perform profiling of epigenetic-associated ncRNAs. Analogous to the types of gene regulatory elements bearing distinctive histone and DNA modifications, we developed a bioinformatics method to classify the putative biological functions of ncRNAs based on their enrichment patterns on chromatin with different histone modifications. Our method successfully arranged several well-studied lncRNAs in the correct functional category and predicted functions for hundreds of other ncRNAs from their chromatin association patterns. More importantly, when a similar analysis was performed on multiple cell types, chromatin state-specific ncRNA enrichment patterns were generally conserved, suggesting this is a reliable method for functional classification. Since ncRNA-chromatin interaction is likely a widespread epigenetic regulation mechanism in many cell types, our integrative approach in identifying and classifying chromatin-associated ncRNAs can be broadly applicable to many other cell types to deeper investigate ncRNA functions. However, chromatin association does not guarantee that a ncRNA will have a biological function; furthermore, the histone modification-specific PIRCh-seq approach can only predict putative functions. As such, the true function of each ncRNA still requires further investigation beyond PIRCh-seq.

### RNA secondary structure affects RNA-chromatin interaction

We observed that RNAs attach to chromatin in a domain-specific manner. However, when we surveyed the enriched sites of chromatin-associated RNAs linked to various histone modifications in different cell types, we did not find significant sequence motifs, suggesting the existence of a complex mechanism responsible for the RNA-chromatin interaction. On the other hand, when we integrated PIRCh-seq signals with RNA structural information from previous icSHAPE and RNA modification m^6^A profiles and further evaluated structural information regarding the enriched domains, we found that ncRNAs were likely to bind to chromatin through single-stranded region or bases with m^6^A methylation. This may possibly be explained by the supposition that RNA-dependent recruitment of transcriptional activators and repressors may occur within a double-stranded structural region, and therefore, single-stranded regions are made more accessible to chromatin. In addition, chromatin interactions may also be allele specific, especially when certain alleles result in distinct RNA secondary structures. In conclusion, when taken as a whole, these results open new avenues of inquiry and require further investigation to fully elucidate the molecular mechanisms of ncRNA-chromatin interaction.

## Conclusions

We developed a PIRCh-seq approach that enables to identify chromatin-associated RNAs in a histone modification-specific manner transcriptome-wide. We identified hundreds of chromatin-associated RNAs in several cell types and predicted their putative functions in gene regulation. We found structural preference of RNA bases accessible to chromatin and discovered hundreds of allele-specific RNA-chromatin interactions. We expect broad application of PIRCh-seq to elucidate the basic mechanisms of chromatin-RNA interaction.

## Methods

### Cell culture

V6.5 mouse ES cells were cultured on 0.2% gelatin-coated plates at 37 °C with mES media: 500 ml Knockout DMEM (Gibco), 90 ml FBS, 6 ml non-essential amino acid (NEAA, 100×, Gibco), 6 ml glutamine or glutamax (200 mM stock solution), 6 ml Pen/Strep, 1 ml BME, and 60 μlLIF (Millipore, ESG1106). Mouse embryonic fibroblast (MEF) cells were cultured at 37 °C and 5% CO2 in 450 ml DMEM, 50 ml FBS, 5 ml Pen/Strep, 5 ml NEAA, 5 ml pyruvate, and 4 μl beta-mercaptoethanol. Mouse Neural Precursor cells (NPCs) were cultured in N2B27 medium (DMEM/F12 (Invitrogen, 11320-033), Neurobasal (Gibco, 21103-049), NDiff Neuro-2 Medium Supplement (Millipore, SCM012), B27 Supplement (Gibco, 17504-044)) supplemented with EGF and FGF (10 ng/ml, each) (315-09 and 100-18B, Peprotech). Cells were passaged using Accutase (SCR005, Millipore) and cultured on 0.2% gelatin-coated plates. H9 human embryonic stem cells were seeded in a feeder-free system using Matrigel hESC-Qualified Matrix (*354277*, Corning) and were maintained in Essential 8 media (A1517001, Thermo Fisher Scientific) as described previously [69]. Cells were passaged every 3 days as clumps with 0.5 mM EDTA. Human Female Fibroblasts (HFF) were cultured at 37 °C and 5% CO2 in DMEM supplemented with 1% pen/strep and 10% FBS.

### PIRCh-seq library preparation

To harvest the cells for PIRCh-seq, approximately 4 × 10^7^ cells were trypsinized and pooled into a 50-ml falcon tube, after washing with 40 ml of cold PBS once. Fresh 1% glutaraldehyde in room temperature PBS was created from 25% stock, and the remaining stock was discarded. The cell pellet was resuspended in 1 ml of glutaraldehyde solution, and a p1000 pipette was used to resuspend cells and to top up to 40 ml (1 ml 1% glutaraldehyde/1 million cells). After inverting several times, the tube was gently shaken for 10 min, and then quenched with 1/10 volume of 1.25 M glycine. The tube was inverted several times, shaken gently for 5 min, and spun down 2000*g* for 4 min. The pellet was then washed once with 40 ml cold PBS. The pellet was responded in 1 ml/20million cells of cold PBS. Cells were aliquoted at 1 ml each to a fresh Eppendorf tube and spun down 2000*g* for 4 min. After supernatant was carefully aspirated, cell pellets were flash frozen and stored at − 80 °C if necessary.

For sonication, prepared cell pellets were spun down at 2000*g* for 4 min and any remaining PBS was removed. Lysis per 20 million cells was performed with 1 ml of lysis buffer (1% SDS, 50 mM Tris 7.0, 10 mM EDTA, 1 mM PMSF, 0.1 U/μl Superase-in (Ambion), 1× Proteinase inhibitor (Roche)). Lysate was then sonicated till the chromatin size was ~ 300–2000 bp and the lysate was clear. We used the Covaris E220 equipment with the following settings: fill level 10, duty cycle 15, PIP 140, and cycles/burst 200. In terms of time, we sonicated 20-min pulses to test the optimal time to generate chromatin size ~ 300–2000 bp. The lysates were spun down at 16,000*g* for 10 min. Supernatants were flash frozen and stored at − 80 °C if necessary.

For PIRCh-seq library construction, chromatin was thawed and 10 μl was taken as input. Two hundred microliters was aliquoted per reaction, and 400 μl dilution buffer was added to each reaction. H3 or a specific histone modification antibody was then added (dilution buffer: 0.01% SDS, 1.1% Triton X 100, 1.2 mM EDTA, 16.7 mM Tris 7.0, 167 mM NaCl, 1 mM PMSF, 0.1 U/μl Superase-in (Ambion), 1× Proteinase inhibitor (Roche)). The reaction was shaken end to end at 4 °C overnight. Fifty microliters of Protein A dynabeads was used per 5 μg antibody IP. Beads were washed with 5 times the original volume of dilution buffer 4 times. Notice that it is important not to exceed 200 μl original volume of beads per tube. During the last wash, beads were aliquoted to 1 tube per reaction. The buffer was aspirated, and 200 μl of the IP sample was used to resuspend and transfer beads to the IP sample. The reaction was shaken end to end at room temperature for 2 h. The beads were then washed with 1 ml wash buffer 4 times and resuspended in 50 μl IP elution buffer (1% SDS, 50 mM NaHCO3). The reaction was then vortexed at setting 1 for 15 min. The supernatant was then transferred to a fresh tube, and the bead elution was repeated. The supernatant was combined for a total of 100 μl. Five microliters of 3 M NaOAc was immediately added to neutralize pH. Ten microliter TurboDnase buffer and 1 μl TurboDnase (Ambion) were added, and the reaction was incubated at 37 °C for 30 min. Three microliters of 500 mM EDTA was added to eliminate divalent ions. Five microliters of Proteinase K (Ambion) was added, and the reaction was incubated at 50 °C for 45 min.

To make our sequencing libraries, we extracted RNA using Trizol/chloroform and precipitated the RNA with an equal volume of isopropanol. RNA pellet was washed in 1 ml 70% EtOH, and pellets were resuspended in 10 μl H2O. One microliter of TurboDnase buffer was added, followed by 1 μl TurboDnase, and the reaction at 37 °C for 30 min. 1.2 μl of TurboDnase inactivating reagents were added. The reaction was vortexed for 3 min and spun down. The 10-μl supernatant was heated at 75 °C for 10 min to kill DNase. The reaction was purified using a Nugen Ovation v2 kit and eluted in 5 μl for library preparation.

### ChIRP-seq library preparation

To determine the genome-wide localization of *lnc-Nr2f1*1, we followed protocols previously described [33]. ChIRP was performed using biotinylated probes designed against mouse lnc-Nr2f1 using the ChIRP probes designer (Biosearch Technologies). Independent even and odd probe pools were used to ensure lncRNA-specific retrieval as protocols previously described [25]. “Even” and “odd” sets of probes shared no overlapping sequences, as we performed two independent ChIRP-seq experiments with these two sets of probes separately. Two sets of data were then combined for downstream analysis (see below). Mouse NPC samples are crosslinked in 3% formaldehyde. RNase pre-treated samples are served as negative controls for probe-DNA hybridization. ChIRP libraries are constructed using the NEBNext DNA library preparation kit (New England Biolabs). Sequencing libraries were barcoded using TruSeq adapters and sequenced on HiSeq or NextSeq instruments (Illumina).

### Experimental validation of antibody specificity after glutaraldehyde crosslinking using modified mononucleosomes with barcodes

To ensure that chemical crosslinking with glutaraldehyde did not affect antibody specificity, we followed previous study to test antibody specificity using SNAP-ChIP [26]. During IP pulldown, 15 μl of recombinant nucleosomes (SNAP-ChIP, EpiCypher, 19-1001) was fixed with fresh 1% glutaraldehyde. One percent glutaraldehyde was prepared on the same day in room temperature PBS from 25% stock. Fixation was performed for 10 min at room temperature with gentle shaking. The reaction was then quenched with 1/10 of the original reaction volume of 2.5 M glycine. Tubes were then inverted several times and incubated for 5 min at room temperature with gentle shaking.

Five hundred microliters of fixed chromatin was then added to each tube and pipetted up and down several times to mix well. Ten microliters of nucleosomes mixed with chromatin was taken out of each tube to be used as input during the qPCR. One tablet of Roche complete protease inhibitor was dissolved (Roche, 11697498001) in 50 ml of DI water to obtain a working solution of 50× protease inhibitor cocktail. Sixty microliters of 50× protease inhibitor was added to 3 ml of blank dilution buffer (0.01% SDS, 1.1% Triton X100, 1.2 mM EDTA, 16.7 mM Tris pH 7.0, 167 mM NaCl). One milliliter of dilution buffer with protease inhibitor was then added to each reaction. Five micrograms of appropriate detection antibody for IP pulldown was added to 300 μl of chromatin mixed with crosslinked nucleosomes for each condition. Samples were then incubated at 4 °C overnight with end-to-end shaking.

IP product was eluted as specified during PIRCH library construction. DNA of interest was purified using a Zymo DNA Clean and Concentrator-5 kit (Zymo Research, D4013). The qPCR reaction was performed using Roche’s LightCycler and Brilliant II SYBR® Green QRT-PCR Master Mix (Agilent). We analyzed enrichment for target histone modifications by amplifying unique DNA barcodes at the 3′ end, using primer sequences provided by EpiCypher.

### RT-qPCR

For qRT-PCR analysis, we used Roche’s LightCycler and Brilliant II SYBR® Green QRT-PCR Master Mix (Agilent).

### PIRCh-seq data alignment

Raw reads were uniquely mapped to mm9/hg19 using Tophat with default parameters [70]. Samtools and BedTools were used to transform the mapped bam file into bedGraph and bigwig files for visualization on the UCSC genome browser [71, 72]. RPKM and raw read count for each gene were calculated by self-designed scripts with ensemble annotation, Homo_sapiens.GRCh37.75.gtf for human and Mus_musculus.NCBIM37.67.gtf, and a number of previous publications for mouse samples, respectively [73]. The PIRCh-seq read counts in each sample were then normalized as if the total sequencing depth was 10 million.

### Calculate exon/intron ratio to estimate nascent transcripts

To compare the exon/intron ratios between the PIRCh-seq profiles and other chromatin-associated RNA detection technologies, we aligned raw reads to the same hg19 genome index with Tophat and calculated the reads mapped to intron/exon with ensemble annotation gtf file as described above [70]. For the average read counts around introns, three steps were taken: (1) scaled every intron based on its length, and extended 1 exon length up- and downstream of the selected intron; (2) divided the entire region to 300 windows, and calculate the average number of read counts mapped in each window and then take log2 to scale down the values to avoid interferences from the outliers; and (3) take average for all the windows among all introns. To estimate the correlation between the histone modification-specific PIRCh-seq profile with its corresponding ChIP-seq signals, we obtained ChIP-seq profiles of each histone modification in mESC from ENCODE. And then, for each expressed gene in mESC, the histone modification ChIP-seq signal over input on the gene exon were calculated as the ChIP signal for that gene, and were compared with the corresponding PRICh-seq enrichment score with the same histone modification, and our results indicated that there was no significant correlation with these two sets of signals.

### Gene set enrichment analysis

GSEA software was downloaded from (http://software.broadinstitute.org/gsea/index.jsp) at the Broad Institute website and was utilized to perform the significant differential chromatin enrichment from PIRCh-seq against ncRNA versus coding genes [42]. The ncRNA set consisted of the annotated snoRNA, snRNA, rRNA, lncRNA, miRNA, and miscRNA.

### Data normalization and identification of the chromatin-enriched RNAs

The chromatin-enriched ncRNAs were identified through the limma algorism in R [41]. First, a data matrix was obtained, where each raw read was a gene and each column a sample, and the element of the matrix represented the number of raw reads from PIRCh-seq experiments and inputs. To filter low-express gene, “filterByExpr” method in edgeR [40] was applied as limma algorism recommend. The filtered values in this matrix were then normalized by the limma-voom method in R. After that, differential analysis was performed using the limma gene-wise linear model for each pair of PIRCh replicates over inputs. Non-coding RNAs with *P* value< 0.05 and log2 fold change over inputs > 0 were defined as chromatin enriched. We obtained 258 chromatin-enriched ncRNAs in mouse V6.5 cell line, 200 in MEF, and 110 in NPC. Variation score of each gene was defined as the standard deviations of the fold change among all histone modification-specific PIRCh-seq profiles. The Pearson correlation coefficients between each two PIRCh-seq experiments were calculated, and unsupervised clustering of the correlation matrix was performed in cluster.

### Computational validation of the PIRCh-seq-enriched ncRNAs

In order to validate the PIRCh-enriched candidates by similar methods, we examined 96 published chromatin-association datasets from ChIRP/CHART/RAP/GRID-seq experiments collected by the LnChrom database [43]. We found a total of 23 expressed lncRNAs in the LnChrom database, including *Xist*, *Firre*, *Rmrp*, and *Tug1*, and all of them were positively enriched in our PIRCh experiment and 14 of which were significant with *P* value< 0.05, suggesting the high sensitivity of the PIRCh approach in identifying chromatin-associated lncRNAs. Furthermore, we obtained the genomic binding sites (peaks) of 23 lncRNAs from the aforementioned experiments, and overlapped them with the histone ChIP-seq peaks [37] and got a ratio of the overlap for each lncRNA. We then calculated the Spearman correlation coefficients of these ratios with their corresponding lncRNA’s PIRCh-seq enrichment scores in the same cell line (normalized by the total number of different ChIP-seq peaks), and found that these correlations were significantly higher than random permutations. Peak calling was performed by MACS2 [74] with FDR < 0.05.

### The chromatin association states of the enriched ncRNAs

To cluster chromatin-enriched ncRNAs in distinct groups for functional prediction, we performed t-SNE and *K*-means clustering on the PIRCh enrichment score matrix with the chromatin-associated ncRNAs. The proper *K* number (*K* = 6) was determined by silhouette score [49].

### Nearby coding gene expression comparison

To further evaluate the functional prediction for chromatin-enriched ncRNAs, we first grouped chromatin-enriched ncRNAs by functional classification and then obtained lists of the nearby (± 100 Kb) coding genes. We then calculated the gene expressions of these coding genes and represented them in box plots. Similarly, we obtained a different list of nearby coding genes if the chromatin-enriched ncRNAs were classified based on their chromatin enrichment scores on each histone modification. The significance between each group was estimated by two-tailed Welch’s *T* test.

### lnc-Nr2f1 ChIPR-seq analysis

To further validate the PIRCh-seq candidates, we performed ChIRP-seq on one of the H3K4me3-modified PIRCh-seq-enriched lncRNAs named *lnc-Nr2f1*. Experimental methods were mentioned above, where independent “even” and “odd” probe sets were applied. LncRNA *lnc-Nr2f1* ChIRP-seq data were then analyzed by applying a previously published pipeline [25], where the read alignment was performed in bowtie2 and peak calling in MACS2. Signals from even and odd ChIRP-seq profiles were then merged to reduce false positive caused by probes. We confirmed that *lnc-Nr2f1*-associated genomic regions were indeed enriched with H3K4me3 but no other modifications in NPCs, where the NPC ChIP-seq data was obtained from GSE117289, indicating the high specificity of our PIRCh-seq approach.

### Allelic-specific enrichment analysis in NPC

We first built the CAST/EiJ and 129S1/SvImJ reference genome. The vcf files containing the SNPs in the CAST and 129S1 strains were downloaded from the dbSNP database with the mm9 assembly [75]. Their corresponding genome fasta file was made by GATK toolkit FastaAlternateReferenceMaker and SelectVariants tools [76]. After that, the inputs and PIRCh-seq data in were re-aligned against the CAST and 129S1 indexes by TopHat2 with 0 mismatch (parameter -N 0) to improve the specificity [70]. The allele-specific alignment files were then converted to the bedGraph and bigWigs format using BEDtools. For each gene, its allele-specific expression and enrichment analysis was performed for every SNP on the list, and estimated the significance between CAST and 129S1 through the Mann-Whitney-Wilcoxon test, and *P* value < 0.05 was defined as significant.

### Enriched peak calling from PIRCh-seq profiles

To further investigate the underlying mechanism of RNA-chromatin association, we performed peak calling on PIRCh-seq profiles to identify the bases on each enriched RNA that were mostly affiliated with histone proteins. We first merged data from two replicates of each gene to minimize the experimental deviation bias, and smoothed the normalized read counts on each base through a 5-bp sliding window, along with a 2-bp step size. Peak calling was performed on the smoothed signal with a homemade script. We defined a peak in the local maximum that is fivefold or more amplified relative to the median read counts of the transcript. Next, we applied a bootstrap method by randomly sampling 1000 times with reads from the transcripts, and then estimated the *P* value of each peak as the percentage of cases that were more enriched than observed. Finally, we calculated the relative fold change of each peak with respect to the input control. Significant peaks were filtered based on fold change and *P* value. Finally, RNA structural and modification information was integrated with PIRCh-seq peaks for downstream analysis.

### icSHAPE analysis and structural prediction using RNAfold

To estimate the structure information around PIRCh peak, we integrate mouse V6.5 icSHAPE data from previous paper [61, 65]. Each transcript’s icSHAPE score was calculated by the original icSHAPE pipeline with default parameter. We used homemade script to count icSHAPE score around PIRCh peak (± 200 bp) among all transcripts, and the significance between histone-modification PIRCh peak and random background region was estimated by two-tailed Welch’s *T* test. In terms of *Gas5* in NPC, the structure information of 129S1 allele was represented by V6.5 icSHAPE data, since they have the same sequence. Structure prediction of 129S1 allele and CAST allele was performed by RNAfold web server with default parameter [62]. For 129S1 allele, the higher icSHAPE score at peak region indicates single strand structure, which is similar to the structure prediction from RNAfold. Furthermore, structure prediction of CAST allele of *Gas5* in NPC shows that riboSnitches around PIRCh peak might be the cause of the allele-specific enrichment of *Gas5*s in NPC.

### Statistics

For data presented in Fig. 1b (RT-PCR), *P* values were calculated via the Mann-Whitney-Wilcoxon test in Python. For data presented in Fig. 2d and Additional file 1: Figure S6B (GSEA), enrichment score, *P* values, and FDR were calculated in GSEA. For data presented in Additional file 1: Figure S2G, binomial *P* values were calculated by GREAT. For all *T* test presented in this paper, including Fig. 2e, f, i; Fig. 3f; Additional file 1: Figure S5C; and Fig. 5d–g, *P* values were calculated via two-tailed Welch’s *T* test in Python. For data presented in Fig. 4f, *P* values were calculated via the chi-square test.

## Supplementary information

**Additional file 1:** **Figure S1.** Quality control of histone modification specific PIRCh-seq experiments on distinct cell types. **Figure S2.** PIRCh-seq enriches ncRNAs associated with chromatin. **Figure S3.** PIRCh-seq captures low nascent transcription. **Figure S4.** ncRNAs are more enriched on chromatin than protein coding genes. **Figure S5.** The chromatin-RNA association of ncRNAs give a hint of cis regulation. **Figure S6.** Pattern of ncRNA chromatin association is generally conserved in distinct cell types. **Figure S7.** Allele specific RNA secondary structure and chromatin enrichment of lncRNA Gas5. **Figure S8.** RNA m6A methylation affects chromatin-RNA association.

**Additional file 2 Table S1.** Chromatin enriched ncRNAs in all mouse samples.

**Additional file 3 Table S2.** Allele specific chromatin enrichment in NPC.

**Additional file 4.** Review history.

## Data Availability

The PIRCh-seq and ChIRP-seq data generated in this study is available from NIH GEO with the accession number GSE119006 [77]. All the in house-developed codes/scripts were uploaded to Github website (https://github.com/QuKunLab/PIRCh) [78]. Other published datasets used in this study are listed as follows: (1) GSE69143: mouse *7sk* ChIRP-seq profile [45]; (2) GSE102518: mouse V6.5 ESC ChIP-seq data of H3K4me1, H3K4me3, H3K27ac, H3K27me3, and H3K9me3 [37]; (3) GSE117289: mouse NPC ChIP-seq data of H3K4me1, H3K4me3, H3K27ac, and H3K27me3 [79]; (4) mouse V6.5 ESC icSHAPE data from the whole cell [61]; GSE64169 and cell compartments [65] (GSE117840); (5) GSE52681: mouse ESC m^6^A sequencing data [68]; (5) GSE82312: GRID-seq profiles from human ES cell lines MM1S & MDA231 and mouse ESC [20]; (6) GSE92345: MARGI profiles from human ES cell lines H9 [21]; (7) GSE66478: biochemical fractionation of HEK293 nuclei and RNA-seq of chromatin-associated and soluble-nuclear RNA [19]; (8) GSE21227: chromatin-associated RNAs (CARs) from human fibroblast (HF) cells [17]; (9) GSE57231: total RNA-seq profiles of mouse V6.5 ESC [80]; (10) GSE32916: subcellular RNA-seq profiles of mouse V6.5 ESC [18]; (11) All RNA binding peaks in ChIRP/CHART/RAP/GRID-seq experiments were downloaded from LnChrom [43].

## References

[1] Rinn JL, Chang HY (2012). Genome regulation by long noncoding RNAs. Annu Rev Biochem.

[2] Fu X-D (2014). Non-coding RNA: a new frontier in regulatory biology. Natl Sci Rev.

[3] Khalil AM, Guttman M, Huarte M, Garber M, Raj A, Rivea Morales D, Thomas K, Presser A, Bernstein BE, van Oudenaarden A (2009). Many human large intergenic noncoding RNAs associate with chromatin-modifying complexes and affect gene expression. Proc Natl Acad Sci U S A.

[4] Flynn RA, Chang HY (2014). Long noncoding RNAs in cell-fate programming and reprogramming. Cell Stem Cell.

[5] Djebali S, Davis CA, Merkel A, Dobin A, Lassmann T, Mortazavi A, Tanzer A, Lagarde J, Lin W, Schlesinger F (2012). Landscape of transcription in human cells. Nature.

[6] Liu SJ, Horlbeck MA, Cho SW, Birk HS, Malatesta M, He D, Attenello FJ, Villalta JE, Cho MY, Chen Y (2017). CRISPRi-based genome-scale identification of functional long noncoding RNA loci in human cells. Science.

[7] Sauvageau M, Goff LA, Lodato S, Bonev B, Groff AF, Gerhardinger C, Sanchez-Gomez DB, Hacisuleyman E, Li E, Spence M (2013). Multiple knockout mouse models reveal lincRNAs are required for life and brain development. eLife.

[8] Xu J, Cao X (2019). Long noncoding RNAs in the metabolic control of inflammation and immune disorders. Cell Mol Immunol.

[9] Tsai M-C, Manor O, Wan Y, Mosammaparast N, Wang JK, Lan F, Shi Y, Segal E, Chang HY (2010). Long noncoding RNA as modular scaffold of histone modification complexes. Science.

[10] West JA, Davis CP, Sunwoo H, Simon MD, Sadreyev RI, Wang PI, Tolstorukov MY, Kingston RE (2014). The long noncoding RNAs NEAT1 and MALAT1 bind active chromatin sites. Mol Cell.

[11] Lai F, Orom UA, Cesaroni M, Beringer M, Taatjes DJ, Blobel GA, Shiekhattar R (2013). Activating RNAs associate with mediator to enhance chromatin architecture and transcription. Nature.

[12] Wang KC, Yang YW, Liu B, Sanyal A, Corces-Zimmerman R, Chen Y, Lajoie BR, Protacio A, Flynn RA, Gupta RA (2011). A long noncoding RNA maintains active chromatin to coordinate homeotic gene expression. Nature.

[13] Zhao J, Ohsumi TK, Kung JT, Ogawa Y, Grau DJ, Sarma K, Song JJ, Kingston RE, Borowsky M, Lee JT (2010). Genome-wide identification of polycomb-associated RNAs by RIP-seq. Mol Cell.

[14] Darnell RB (2010). HITS-CLIP: panoramic views of protein-RNA regulation in living cells. Wiley interdisciplinary reviews RNA.

[15] G Hendrickson D, Kelley DR, Tenen D, Bernstein B, Rinn JL: Widespread RNA binding by chromatin-associated proteins. Genome Biol 2016, 17:674. 10.1186/s13059-016-0878-3 PMC4756407 26883116

[16] Kurup JT, Kidder BL (2018). Identification of H4K20me3- and H3K4me3-associated RNAs using CARIP-Seq expands the transcriptional and epigenetic networks of embryonic stem cells. J Biol Chem.

[17] Mondal T, Rasmussen M, Pandey GK, Isaksson A, Kanduri C (2010). Characterization of the RNA content of chromatin. Genome Res.

[18] Bhatt DM, Pandya-Jones A, Tong A-J, Barozzi I, Lissner MM, Natoli G, Black DL, Smale ST (2012). Transcript dynamics of proinflammatory genes revealed by sequence analysis of subcellular RNA fractions. Cell.

[19] Werner MS, Ruthenburg AJ (2015). Nuclear fractionation reveals thousands of chromatin-tethered noncoding RNAs adjacent to active genes. Cell Rep.

[20] Li X, Zhou B, Chen L, Gou L-T, Li H, Fu X-D (2017). GRID-seq reveals the global RNA-chromatin interactome. Nat Biotechnol.

[21] Sridhar B, Rivas-Astroza M, Nguyen TC, Chen W, Yan Z, Cao X, Hebert L, Zhong S (2017). Systematic mapping of RNA-chromatin interactions in vivo. Current biology : CB.

[22] Quinodoz SA, Ollikainen N, Tabak B, Palla A, Schmidt JM, Detmar E, Lai MM, Shishkin AA, Bhat P, Takei Y (2018). Higher-order inter-chromosomal hubs shape 3D genome organization in the nucleus. Cell.

[23] Margueron R, Reinberg D (2011). The Polycomb complex PRC2 and its mark in life. Nature.

[24] Soibam B (2017). Super-lncRNAs: identification of lncRNAs that target super-enhancers via RNA:DNA:DNA triplex formation. RNA (New York, NY).

[25] Chu C, Qu K, Zhong FL, Artandi SE, Chang HY (2011). Genomic maps of long noncoding RNA occupancy reveal principles of RNA-chromatin interactions. Mol Cell.

[26] Shah RN, Grzybowski AT, Cornett EM, Johnstone AL, Dickson BM, Boone BA, Cheek MA, Cowles MW, Maryanski D, Meiners MJ (2018). Examining the Roles of H3K4 Methylation States with Systematically Characterized Antibodies. Mol Cell.

[27] Simon MD, Pinter SF, Fang R, Sarma K, Rutenberg-Schoenberg M, Bowman SK, Kesner BA, Maier VK, Kingston RE, Lee JT (2013). High-resolution Xist binding maps reveal two-step spreading during X-chromosome inactivation. Nature.

[28] Pandey RR, Mondal T, Mohammad F, Enroth S, Redrup L, Komorowski J, Nagano T, Mancini-DiNardo D, Kanduri C (2008). Kcnq1ot1 antisense noncoding RNA mediates lineage-specific transcriptional silencing through chromatin-level regulation. Mol Cell.

[29] Monnier P, Martinet C, Pontis J, Stancheva I, Ait-Si-Ali S, Dandolo L (2013). H19 lncRNA controls gene expression of the imprinted gene network by recruiting MBD1. Proc Natl Acad Sci.

[30] Royce-Tolland ME, Andersen AA, Koyfman HR, Talbot DJ, Wutz A, Tonks ID, Kay GF, Panning B (2010). The A-repeat links ASF/SF2-dependent Xist RNA processing with random choice during X inactivation. Nat Struct Mol Biol.

[31] Chu C, Zhang QC, da Rocha ST, Flynn RA, Bharadwaj M, Calabrese JM, Magnuson T, Heard E, Chang HY (2015). Systematic discovery of Xist RNA binding proteins. Cell.

[32] Lu Z, Zhang QC, Lee B, Flynn RA, Smith MA, Robinson JT, Davidovich C, Gooding AR, Goodrich KJ, Mattick JS (2016). RNA duplex map in living cells reveals higher-order transcriptome structure. Cell.

[33] Ang CE, Ma Q, Wapinski OL, Fan S, Flynn RA, Lee QY, Coe B, Onoguchi M, Olmos VH, Do BT (2019). The novel lncRNA lnc-NR2F1 is pro-neurogenic and mutated in human neurodevelopmental disorders. eLife.

[34] McLean CY, Bristor D, Hiller M, Clarke SL, Schaar BT, Lowe CB, Wenger AM, Bejerano G (2010). GREAT improves functional interpretation of cis-regulatory regions. Nat Biotechnol.

[35] Simon MD, Wang CI, Kharchenko PV, West JA, Chapman BA, Alekseyenko AA, Borowsky ML, Kuroda MI, Kingston RE (2011). The genomic binding sites of a noncoding RNA. Proc Natl Acad Sci U S A.

[36] Engreitz JM, Pandya-Jones A, McDonel P, Shishkin A, Sirokman K, Surka C, Kadri S, Xing J, Goren A, Lander ES (2013). The Xist lncRNA exploits three-dimensional genome architecture to spread across the X chromosome. Science.

[37] Zviran A, Mor N, Rais Y, Gingold H, Peles S, Chomsky E, Viukov S, Buenrostro JD, Scognamiglio R, Weinberger L (2019). Deterministic somatic cell reprogramming involves continuous transcriptional changes governed by Myc and epigenetic-driven modules. Cell Stem Cell.

[38] Hsieh TS, Fudenberg G, Goloborodko A, Rando OJ (2016). Micro-C XL: assaying chromosome conformation from the nucleosome to the entire genome. Nat Methods.

[39] Zeng PY, Vakoc CR, Chen ZC, Blobel GA, Berger SL (2006). In vivo dual cross-linking for identification of indirect DNA-associated proteins by chromatin immunoprecipitation. Biotechniques.

[40] Robinson MD, McCarthy DJ, Smyth GK (2010). edgeR: a bioconductor package for differential expression analysis of digital gene expression data. Bioinformatics (Oxford, England).

[41] Ritchie ME, Phipson B, Wu D, Hu Y, Law CW, Shi W, Smyth GK (2015). limma powers differential expression analyses for RNA-sequencing and microarray studies. Nucleic Acids Res.

[42] Subramanian A, Tamayo P, Mootha VK, Mukherjee S, Ebert BL, Gillette MA, Paulovich A, Pomeroy SL, Golub TR, Lander ES, Mesirov JP (2005). Gene set enrichment analysis: a knowledge-based approach for interpreting genome-wide expression profiles. Proc Natl Acad Sci U S A.

[43] Yu F, Zhang G, Shi A, Hu J, Li F, Zhang X, Zhang Y, Huang J, Xiao Y, Li X, Cheng S (2018). LnChrom: a resource of experimentally validated lncRNA-chromatin interactions in human and mouse. Database.

[44] Rando OJ, Chang HY (2009). Genome-wide views of chromatin structure. Annu Rev Biochem.

[45] Flynn RA, Do BT, Rubin AJ, Calo E, Lee B, Kuchelmeister H, Rale M, Chu C, Kool ET, Wysocka J (2016). 7SK-BAF axis controls pervasive transcription at enhancers. Nat Struct Mol Biol.

[46] Wang Z, Zang C, Rosenfeld JA, Schones DE, Barski A, Cuddapah S, Cui K, Roh T-Y, Peng W, Zhang MQ, Zhao K (2008). Combinatorial patterns of histone acetylations and methylations in the human genome. Nat Genet.

[47] Ernst J, Kheradpour P, Mikkelsen TS, Shoresh N, Ward LD, Epstein CB, Zhang X, Wang L, Issner R, Coyne M (2011). Mapping and analysis of chromatin state dynamics in nine human cell types. Nature.

[48] Ernst J, Kellis M (2012). ChromHMM: automating chromatin-state discovery and characterization. Nat Methods.

[49] Rousseeuw PJ (1987). Silhouettes: a graphical aid to the interpretation and validation of cluster analysis. J Comput Appl Math.

[50] Hirata H, Hinoda Y, Shahryari V, Deng G, Nakajima K, Tabatabai ZL, Ishii N, Dahiya R (2015). Long noncoding RNA MALAT1 promotes aggressive renal cell carcinoma through Ezh2 and interacts with miR-205. Cancer Res.

[51] Jia J, Li F, Tang X-S, Xu S, Gao Y, Shi Q, Guo W, Wang X, He D, Guo P (2016). Long noncoding RNA DANCR promotes invasion of prostate cancer through epigenetically silencing expression of TIMP2/3. Oncotarget.

[52] Quek XC, Thomson DW, Maag JLV, Bartonicek N, Signal B, Clark MB, Gloss BS, Dinger ME (2015). lncRNAdb v2.0: expanding the reference database for functional long noncoding RNAs. Nucleic Acids Res.

[53] Yan P, Luo S, Lu JY, Shen X (2017). Cis- and trans-acting lncRNAs in pluripotency and reprogramming. Curr Opin Genet Dev.

[54] Kopp F, Mendell JT (2018). Functional classification and experimental dissection of long noncoding RNAs. Cell.

[55] Meshorer E, Yellajoshula D, George E, Scambler PJ, Brown DT, Misteli T (2006). Hyperdynamic plasticity of chromatin proteins in pluripotent embryonic stem cells. Dev Cell.

[56] Bernstein BE, Mikkelsen TS, Xie X, Kamal M, Huebert DJ, Cuff J, Fry B, Meissner A, Wernig M, Plath K (2006). A bivalent chromatin structure Marks key developmental genes in embryonic stem cells. Cell.

[57] Wapinski OL, Vierbuchen T, Qu K, Lee QY, Chanda S, Fuentes DR, Giresi PG, Ng YH, Marro S, Neff NF (2013). Hierarchical mechanisms for direct reprogramming of fibroblasts to neurons. Cell.

[58] Mikkelsen TS, Ku M, Jaffe DB, Issac B, Lieberman E, Giannoukos G, Alvarez P, Brockman W, Kim T-K, Koche RP (2007). Genome-wide maps of chromatin state in pluripotent and lineage-committed cells. Nature.

[59] Voigt P, LeRoy G, Drury WJ, Zee BM, Son J, Beck DB, Young NL, Garcia BA, Reinberg D (2012). Asymmetrically modified nucleosomes. Cell.

[60] Kertesz M, Wan Y, Mazor E, Rinn JL, Nutter RC, Chang HY, Segal E (2010). Genome-wide measurement of RNA secondary structure in yeast. Nature.

[61] Spitale RC, Flynn RA, Zhang QC, Crisalli P, Lee B, Jung J-W, Kuchelmeister HY, Batista PJ, Torre EA, Kool ET, Chang HY (2015). Structural imprints in vivo decode RNA regulatory mechanisms. Nature.

[62] Hofacker IL (2003). Vienna RNA secondary structure server. Nucleic Acids Res.

[63] Wan Y, Qu K, Zhang QC, Flynn RA, Manor O, Ouyang Z, Zhang J, Spitale RC, Snyder MP, Segal E, Chang HY (2014). Landscape and variation of RNA secondary structure across the human transcriptome. Nature.

[64] Sun D, Yu Z, Fang X, Liu M, Pu Y, Shao Q, Wang D, Zhao X, Huang A, Xiang Z (2017). LncRNA GAS5 inhibits microglial M2 polarization and exacerbates demyelination. EMBO Rep.

[65] Sun L, Fazal FM, Li P, Broughton JP, Lee B, Tang L, Huang W, Kool ET, Chang HY, Zhang QC (2019). RNA structure maps across mammalian cellular compartments. Nat Struct Mol Biol.

[66] Roost C, Lynch SR, Batista PJ, Qu K, Chang HY, Kool ET (2015). Structure and thermodynamics of N 6-methyladenosine in RNA: a spring-loaded base modification. J Am Chem Soc.

[67] Liu N, Pan T (2016). N6-methyladenosine–encoded epitranscriptomics. Nat Struct Mol Biol.

[68] Batista PJ, Molinie B, Wang J, Qu K, Zhang J, Li L, Bouley DM, Lujan E, Haddad B, Daneshvar K (2014). m6A RNA modification controls cell fate transition in mammalian embryonic stem cells. Cell Stem Cell.

[69] Chen G, Gulbranson DR, Hou Z, Bolin JM, Ruotti V, Probasco MD, Smuga-Otto K, Howden SE, Diol NR, Propson NE (2011). Chemically defined conditions for human iPSC derivation and culture. Nat Methods.

[70] Trapnell C, Roberts A, Goff L, Pertea G, Kim D, Kelley DR, Pimentel H, Salzberg SL, Rinn JL, Pachter L (2012). Differential gene and transcript expression analysis of RNA-seq experiments with TopHat and Cufflinks. Nat Protoc.

[71] Li H, Handsaker B, Wysoker A, Fennell T, Ruan J, Homer N, Marth G, Abecasis G, Durbin R, Subgroup GPDP (2009). The sequence alignment/map format and SAMtools. Bioinformatics (Oxford, England).

[72] Quinlan AR, Hall IM (2010). BEDTools: a flexible suite of utilities for comparing genomic features. Bioinformatics (Oxford, England).

[73] Guttman M, Garber M, Levin JZ, Donaghey J, Robinson J, Adiconis X, Fan L, Koziol MJ, Gnirke A, Nusbaum C (2010). Ab initio reconstruction of cell type–specific transcriptomes in mouse reveals the conserved multi-exonic structure of lincRNAs. Nat Biotechnol.

[74] Zhang Y, Liu T, Meyer CA, Eeckhoute J, Johnson DS, Bernstein BE, Nusbaum C, Myers RM, Brown M, Li W, Liu XS (2008). Model-based analysis of ChIP-Seq (MACS). Genome Biol.

[75] Sherry ST, Ward M, Sirotkin K (1999). dbSNP-database for single nucleotide polymorphisms and other classes of minor genetic variation. Genome Res.

[76] McKenna A, Hanna M, Banks E, Sivachenko A, Cibulskis K, Kernytsky A, Garimella K, Altshuler D, Gabriel S, Daly M, DePristo MA (2010). The Genome Analysis Toolkit: a MapReduce framework for analyzing next-generation DNA sequencing data. Genome Res.

[77] Chang HY, Fang J, Ma Q, Chu C, Huang B, Li L, Cai P, Batista PJ, Tolentino KEM, Xu J, Li R, Du P, Qu K: Functional classification of chromatin associated lncRNAs via histone modification specific PIRCh-seq analysis. Database. Gene Expression Omnibus. 2019. https://www.ncbi.nlm.nih.gov/geo/query/acc.cgi?acc=GSE119006. Accessed 30 Oct 2019.

[78] Chang HY, Fang J, Ma Q, Chu C, Huang B, Li L, Cai P, Batista PJ, Tolentino KEM, Xu J, Li R, Du P, Qu K: PIRCh. Github. 2019. https://github.com/QuKunLab/PIRCh. Accessed 11 Apr 2019. 10.1186/s13059-019-1880-3 PMC6924075 31862000

[79] Kloet SL, Karemaker ID, van Voorthuijsen L, Lindeboom RGH, Baltissen MP, Edupuganti RR, Poramba-Liyanage DW, Jansen PWTC, Vermeulen M (2018). NuRD-interacting protein ZFP296 regulates genome-wide NuRD localization and differentiation of mouse embryonic stem cells. Nat Commun.

[80] Boutz PL, Bhutkar A, Sharp PA (2015). Detained introns are a novel, widespread class of post-transcriptionally spliced introns. Genes Dev.

