# Supplementary material for: PIRCh-seq: functional classification of non-coding RNAs associated with distinct histone modifications
Source: Genome Biol. 2019 Dec 20;20:292. doi: 10.1186/s13059-019-1880-3 (PMC6924075; doi:10.1186/s13059-019-1880-3)
Supplement: Supplementary file 1 — Additional file 1: Figure S1. Quality control of histone modification specific PIRCh-seq experiments on distinct cell types. Figure S2. PIRCh-seq enriches ncRNAs associated with chromatin. Figure S3. PIRCh-seq captures low nascent transcription. Figure S4. ncRNAs are more enriched on chromatin than protein coding genes. Figure S5. The chromatin-RNA association of ncRNAs give a hint of cis regulation. Figure S6. Pattern of ncRNA chromatin association is generally conserved in distinct cell types. Figure S7. Allele specific RNA secondary structure and chromatin enrichment of lncRNA Gas5. Figure S8. RNA m6A methylation affects chromatin-RNA association. [file 13059_2019_1880_MOESM1_ESM.pdf]

Figure S1

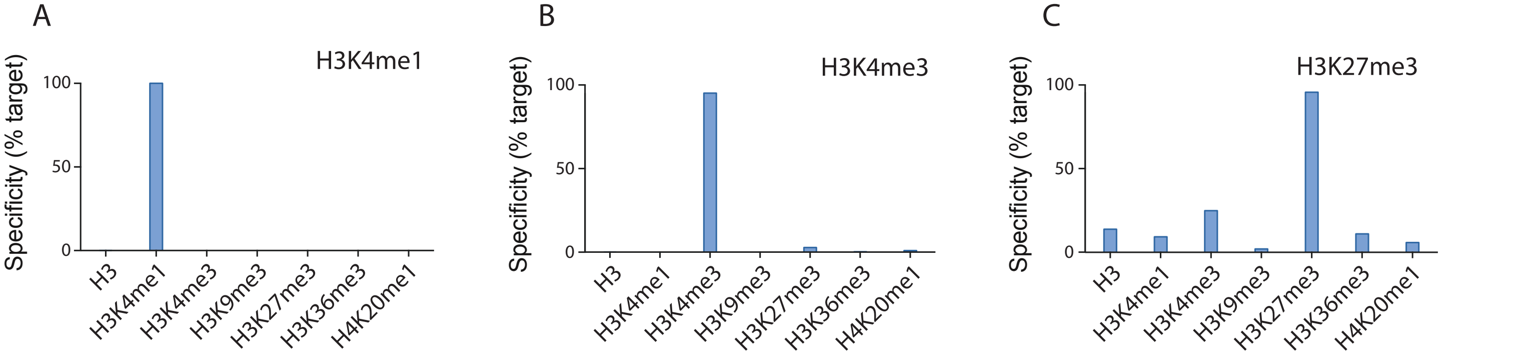

D

Data Summary

	mESC	MEF	NPC	H9	HFF
Input	✓	✓	✓	✓	✓
H3	✓	✓		✓	✓
H3K4me1	✓	✓		✓	
H3K4me3	✓	✓	✓	✓	
H3K27ac	✓	✓	✓		
H3K27me3	✓	✓	✓		
H3K9me3	✓	✓			
H4K16ac	✓	✓			

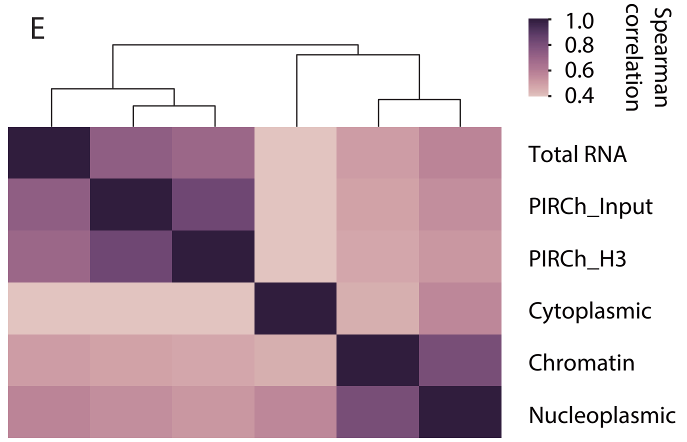

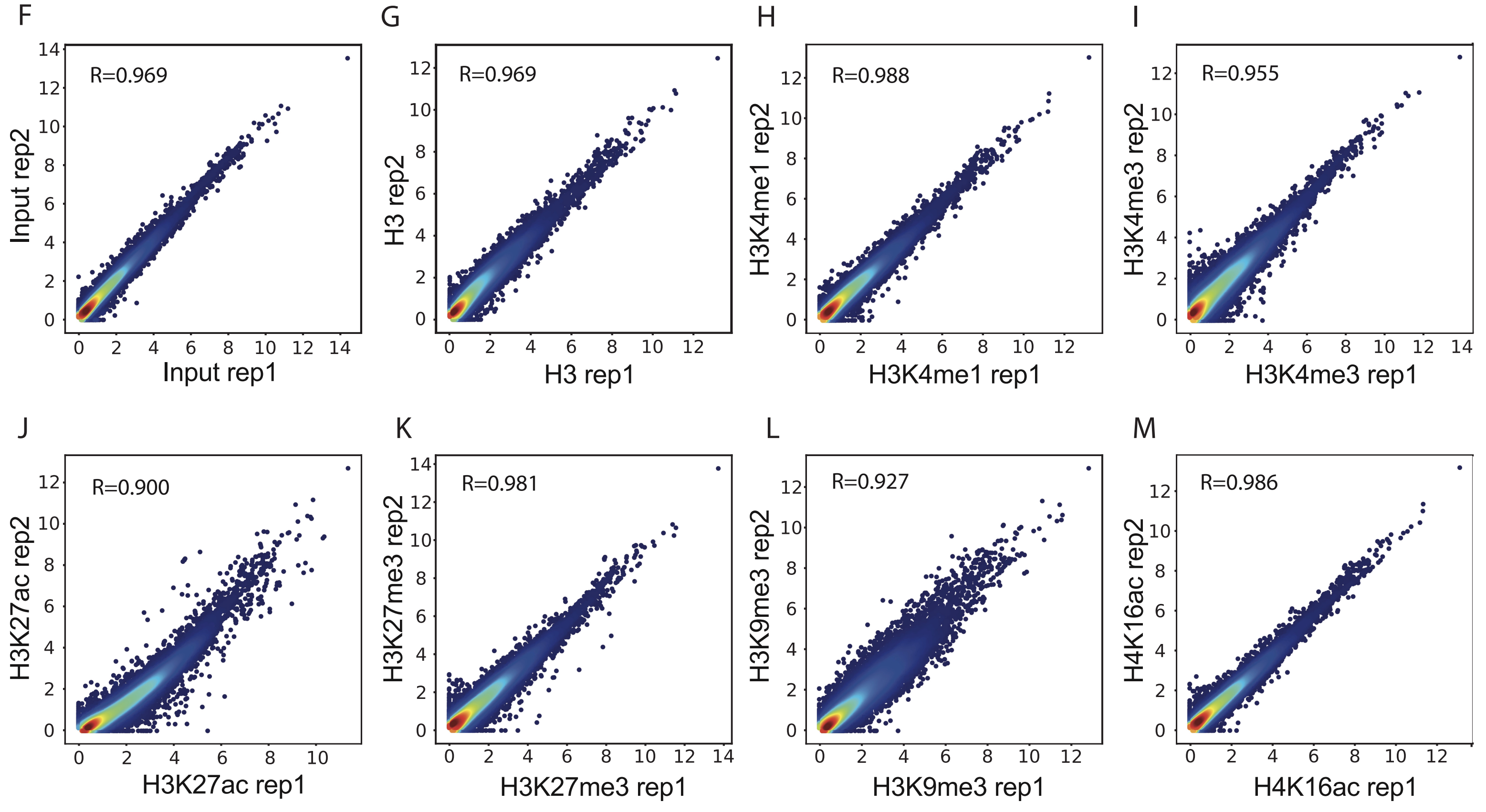

Supplementary Figure 1. Quality control of histone modification specific PIRCh-seq experiments on distinct cell types.
A-C. The specificity of IP of different antibodies H3K4me1 (A), H3K4me3 (B), and H3K27me3 (C) after glutaraldehyde crosslinking usinga modified mononucleosomes with barcodes. 7 different mononucleosomes with barcodes were tested.
D. Table summarizing PIRCh-seq experiments performed in this paper.
E. Spearman correlation heatmap of RNA expressions obtained from total RNA, PRICh-seq input, H3 PIRCh-seq, and RNAs from cytoplasm, chromatin and nucleoplasm.
F-M. Scatter plots of expressed transcripts (log2) in two PIRCh-seq replicates with correlation score R on different histone modification, H3, H3K4me1, H3K4me3, H3K27ac, H3K27me3, H3K9me3, and H4K16ac respectively.

Figure S2

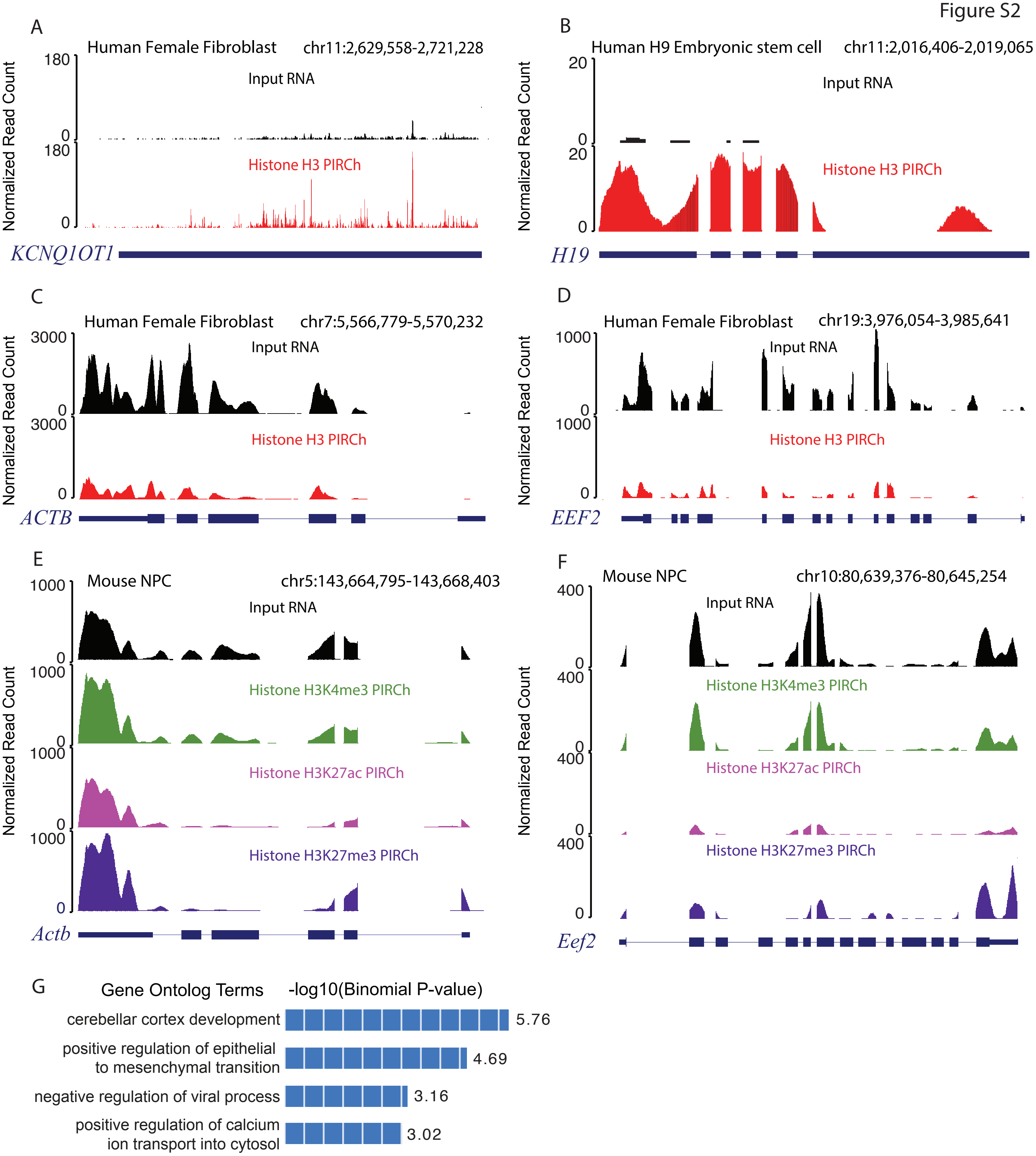

Supplementary Figure 2. PIRCh-seq enriches ncRNAs associated with chromatin

A-B. The overall enrichment of the H3 PIRCh-seq signal (bottom) over input (top) of lncRNA KCNQ1OT1(A, Fold Change = 19) in human female fibroblast cells and H19 (B, Fold Change = 58) in human H9 embryonic stem cells. Normalized read count of input and histone H3 PIRCh-seq signals were shown.

C-D. Normalized read count of input and histone H3 PIRCh-seq signals on protein coding genes ACTB (C), and EEF2 (D) in human female fibroblast cells.

E-F. Normalized UCSC tracks of input and histone modification specific PIRCh-seq signals on protein coding gene Actb (E) and Eef2 (F) in mouse neuronal precursor cells.

G. Top 5 enriched gene ontology of the lnc-Nr2f1 ChIRP-seq peaks using GREAT.

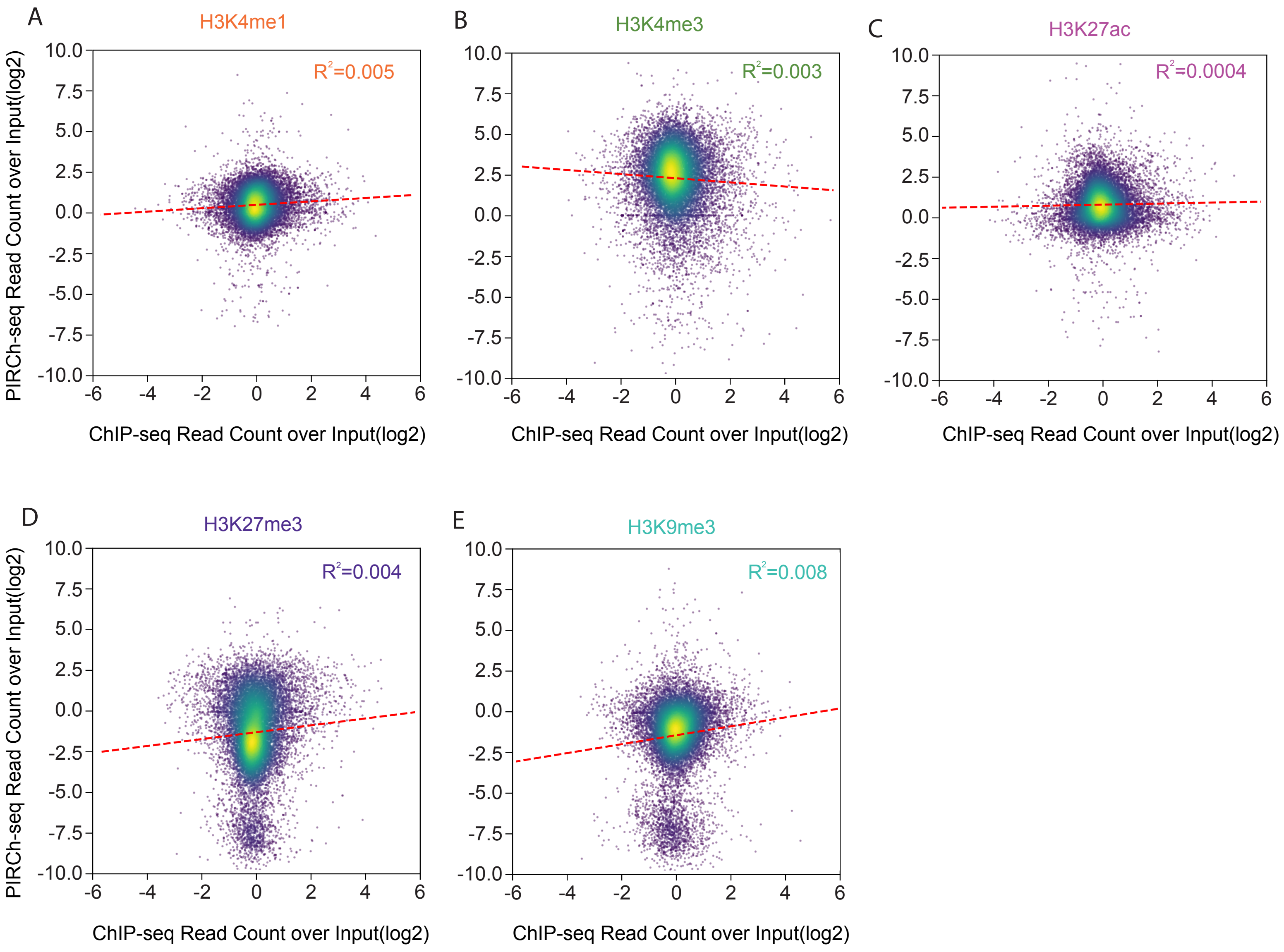

Supplementary Figure 3. PIRCh-seq captures low nascent transcription
A-E. Scatter plot of the PIRCh-seq (y-axis) signal over input vs the corresponding ChIP-seq (x-axis) signal over input for all the expressed genes in mESC, with linear regression (red dotted line). Colors represent the density of point.

Figure S4

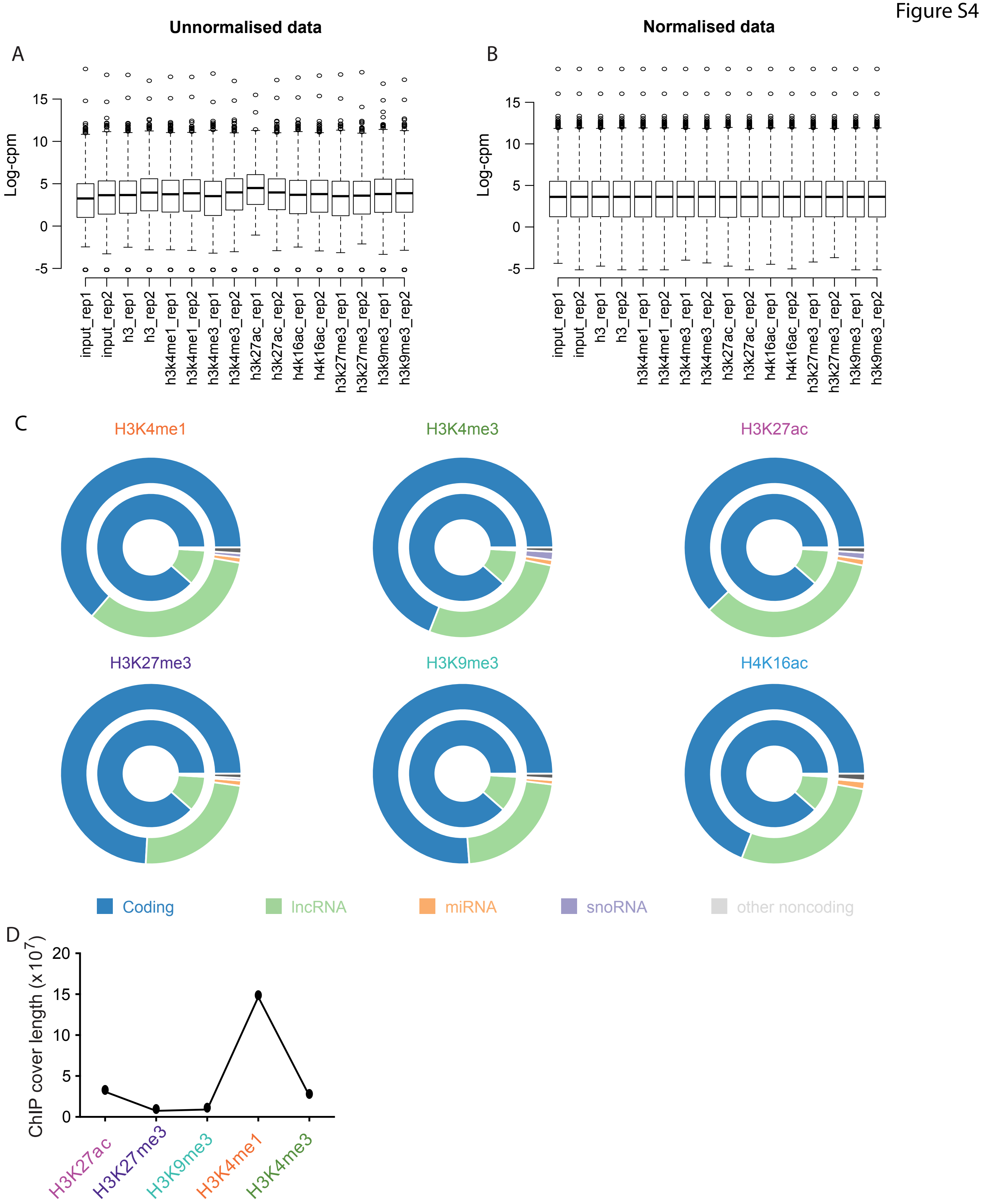

Supplementary Figure 4. ncRNAs are more enriched on chromatin than protein coding genes.
A-B. Box-plots of the PIRCh-seq signal before (A) and after (B) normalization using the limma algorithm in R. cpm represents count per million, and log scale is shown. Center lines represent mean values; box limits represent the interquartile range; whiskers each extend 1.5 times the interquartile range; dots represent outliers.
C. Circle plots showing the distribution of the expressed (inner circle, all obtained from inputs) and PIRCh enriched (outer circle) RNA types associated with different histone modifications. ncRNAs are highly enriched in PIRCh compared with coding genes. Whether an RNA is expressed or not was filtered by the “filterByExpr” module in edgeR based on the RNA expressions in inputs.
D. Total length of the genomic regions (in bp) covered by each histone modification ChIP-seq peak in mESC.

Figure S5

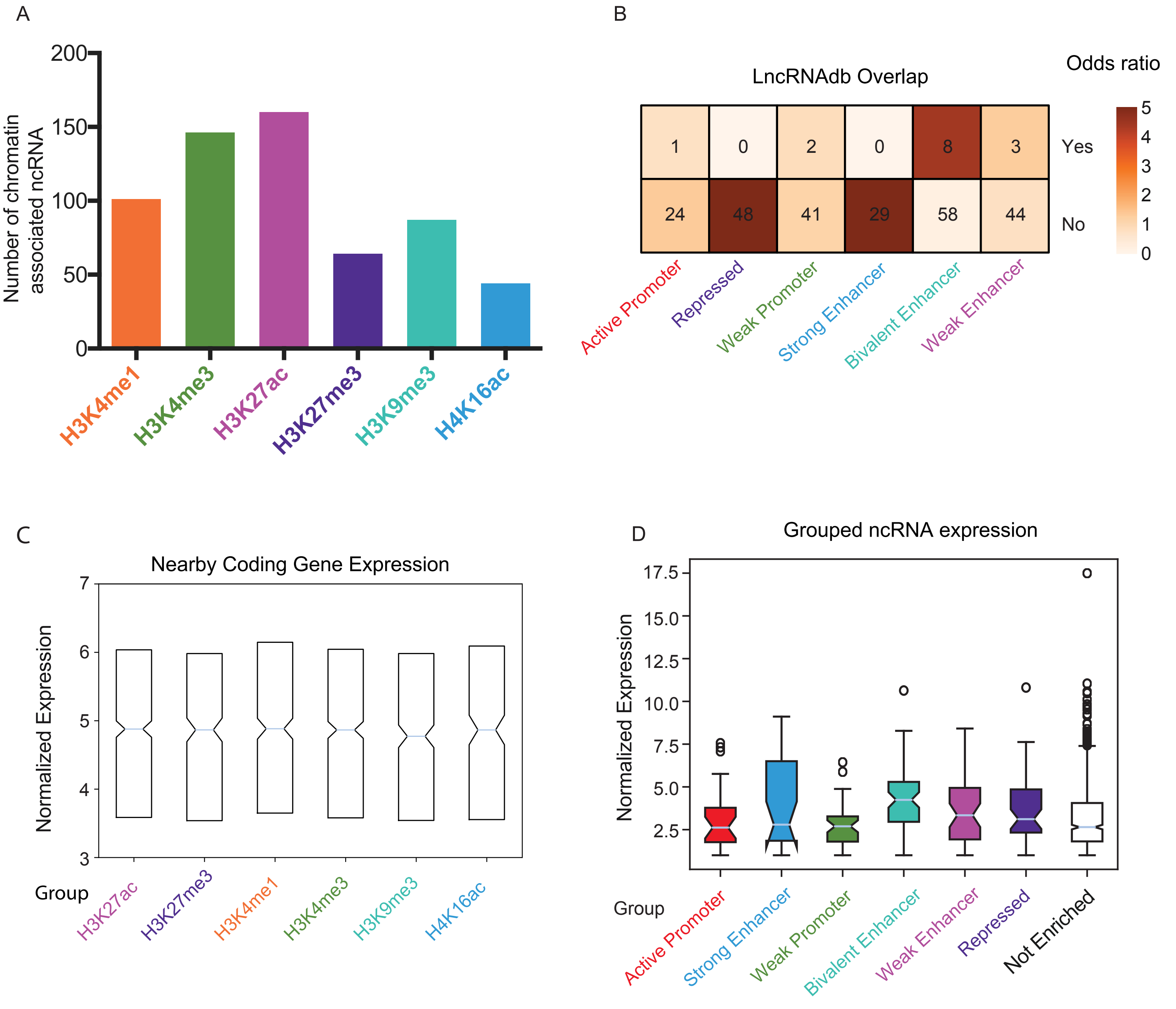

Supplementary Figure 5. The chromatin-RNA association of ncRNAs give a hint of cis regulation.
A. Bar chart showing the number of ncRNAs enriched at chromatin with specific histone modifications in mESC.
B. The odds ratio of the PIRCh enriched ncRNAs overlap with the chromatin enriched ncRNAs defined in lncRNAdb. “Yes” means the ncRNA is both PIRCh enriched and found in lncRNAdb, and “No” means PIRCh enriched but was not identified in lncRNAdb.
C. Box-plot of the expression of the coding genes near (+/-100Kb) each group of histone modification specific PIRCh-seq enriched ncRNAs. Center lines represent mean values; box limits represent the interquartile range. The expression of the coding genes that close to the ncRNAs enriched on active chromatin shows no significant difference between that with repressed chromatin.
D. Box-plot of the expression of each groups of PIRCh cluttered ncRNAs defined in Figure 3D. Center lines represent mean values; box limits represent the interquartile range; whiskers each extend 1.5 times the interquartile range; dots represent outliers.

Figure S6

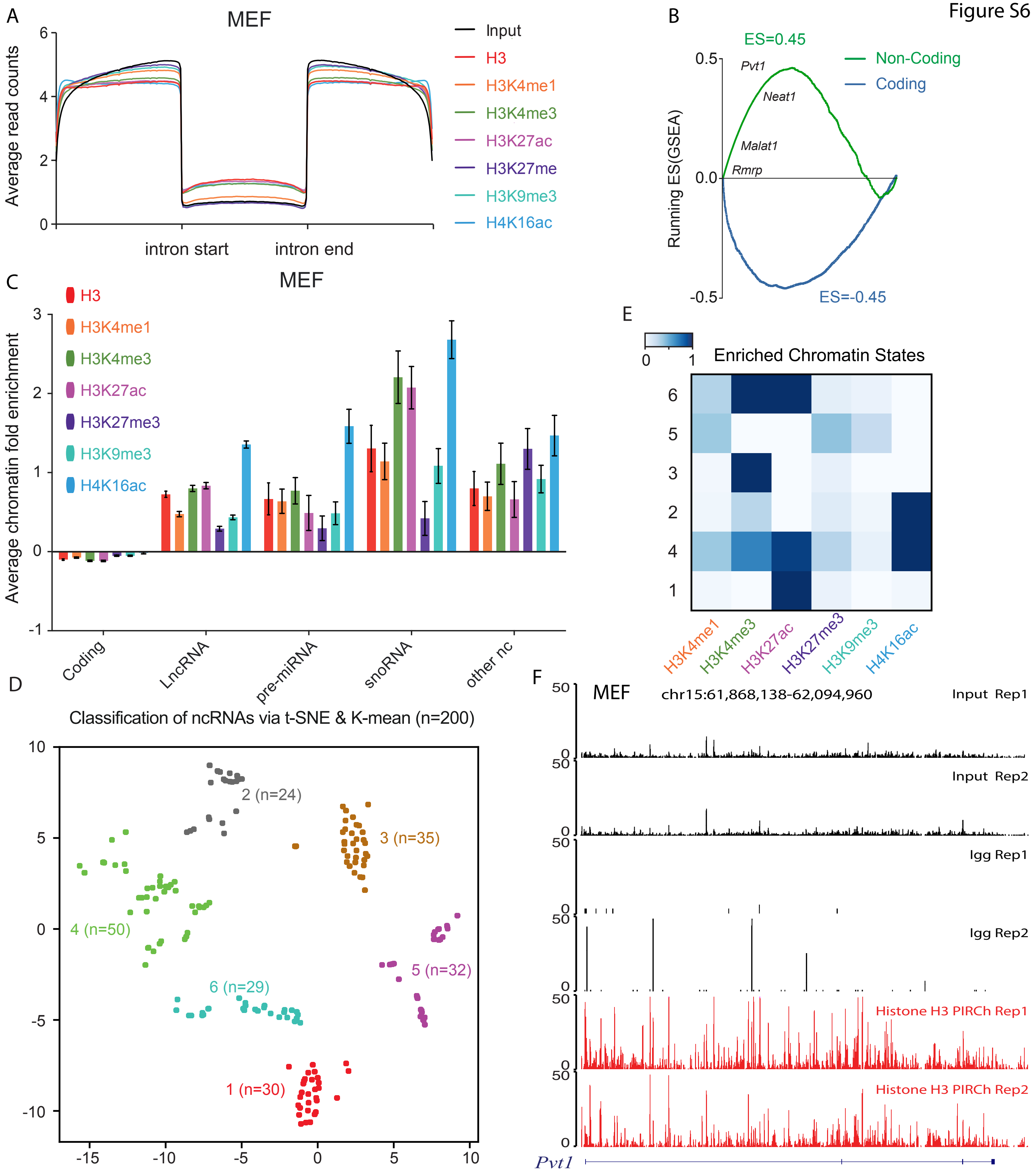

Supplementary Figure 6. Pattern of ncRNA chromatin association is generally conserved in distinct cell types.

A. Normalized average read coverage around introns from histone modification specific PIRCh-seq profiles (colored) and inputs (black) in MEF.

B. Gene set enrichment analysis (GSEA) shows highly statistical enriched (FDR=0, P<0.0001) of non-coding genes (Green) and depleted of coding genes (Blue) on histone H3 in MEF. Genes were ranked by their histone H3 PIRCh enrichment scores.

C. Average fold enrichment (calculated by limma in R) of the coding gene, lncRNA, pre-miRNA, snoRNA and other ncRNA from histone modification specific PIRCh-seq profiles (namely H3, H3K4me1, H3K4me3, H3K27ac, H3K27me3, H3K9me3, and H4K16ac) in MEF. Error bar shows the standard deviation from the mean.

D. Functional classification of histone specific chromatin-RNA association patterns defined by chromHMM algorithm.

E. Classification of the PIRCh-seq identified chromatin associated ncRNAs (n=200) in MEF. Scatter plot shows the t-SNE result on PIRCh-seq enrichment score matrix and annotated by K-means clustering.

F. Normalized input, Igg and H3 PIRCh-seq profiles of lncRNA Pvt1 in MEF.

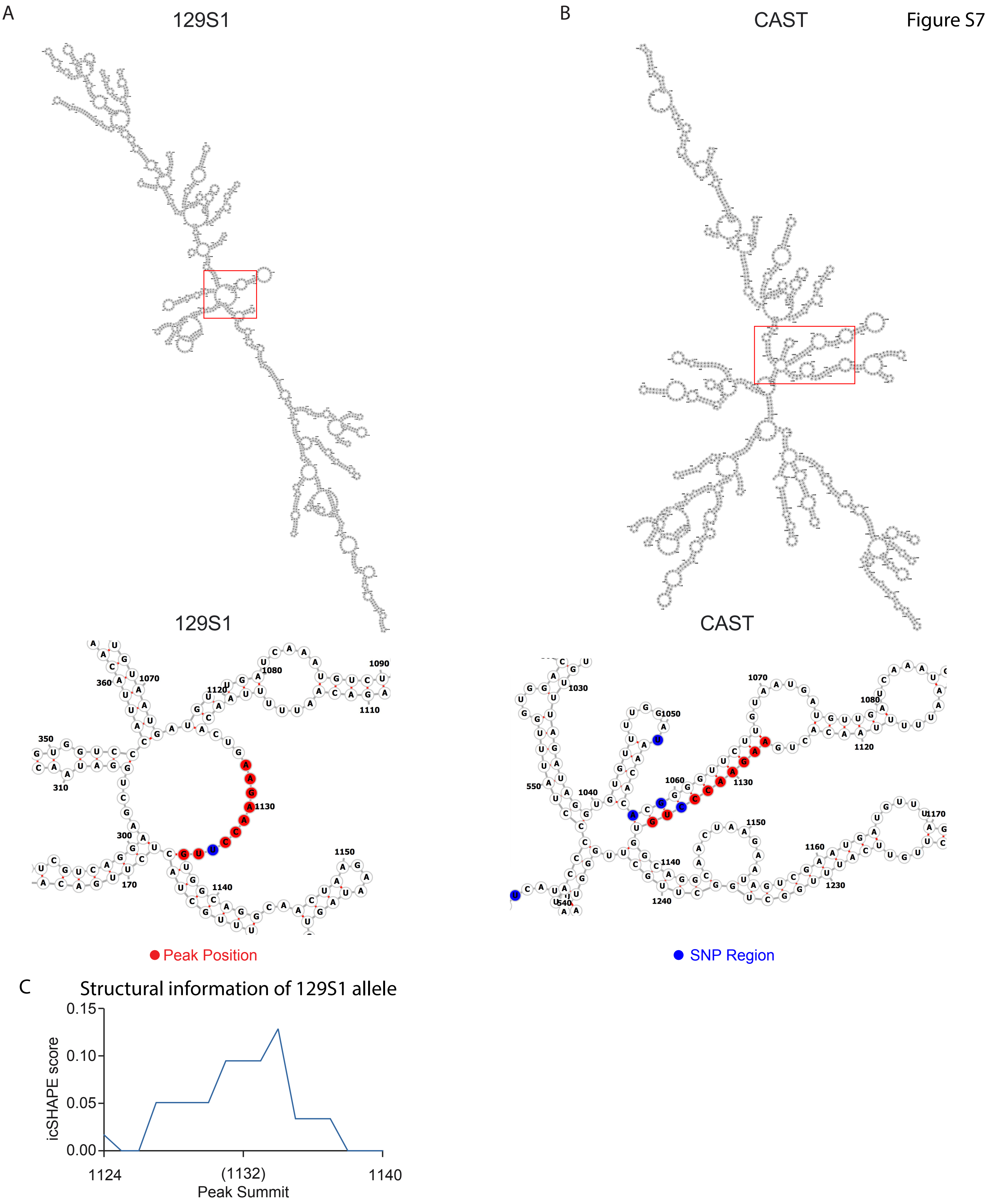

Supplementary Figure 7. Allele specific RNA secondary structure and chromatin enrichment of lncRNA Gas5.
A-B. RNAfold predicted the secondary structure of the 129S1 (A) and CAST (B) allele of Gas5. RiboSNithes are noted in blue and bases attached to chromatin (peaks) are shown in red.
C. Structural information of 129S1 allele around the PIRCh enriched peak region. Data obtained from icSHAPE experiments on mESC.

Figure S8

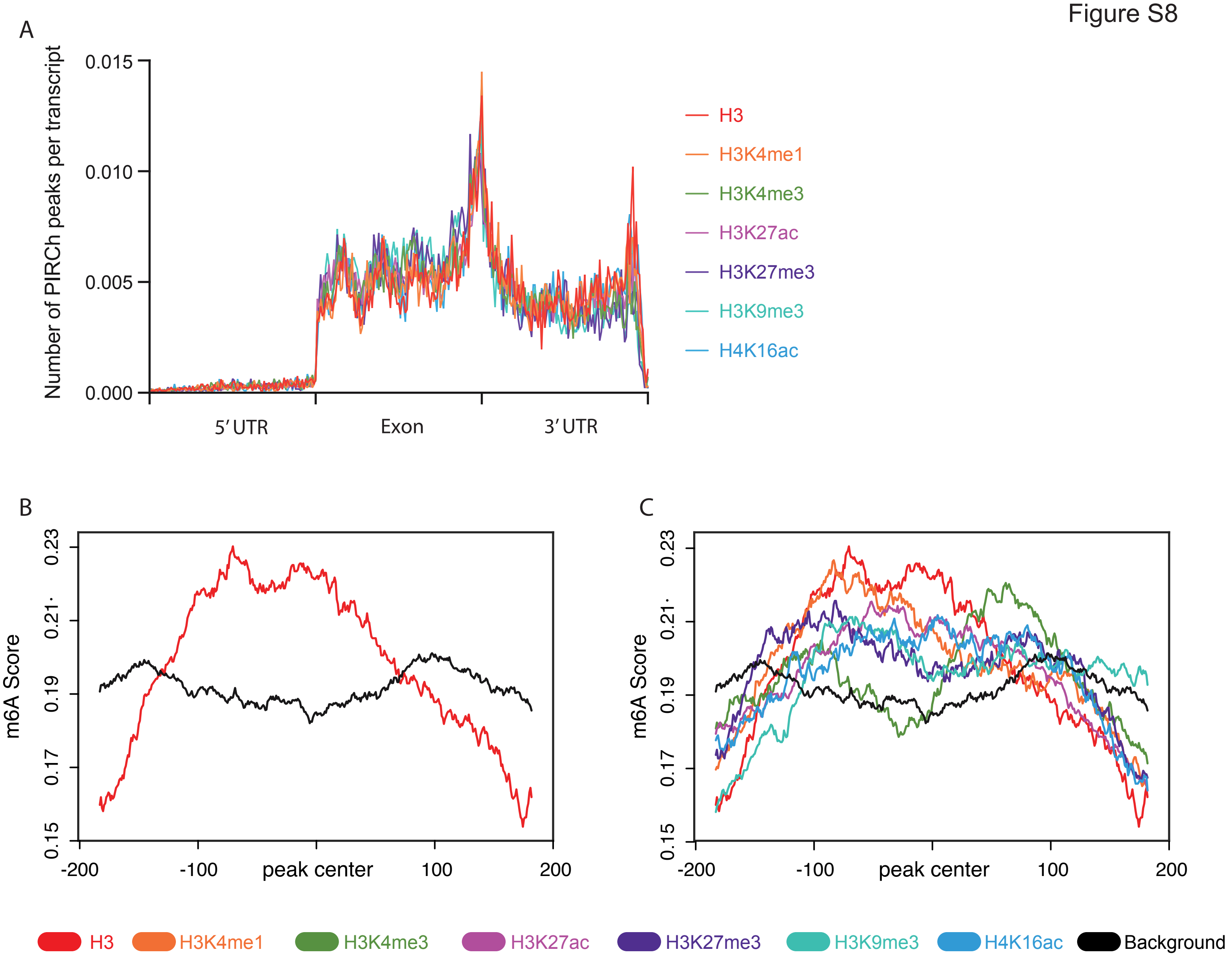

Supplementary Figure 8. RNA m6A methylation affects chromatin-RNA association.
A. Distribution of histone H3 and other chemical modification PIRCh-seq peaks along scaled transcripts.
B-C. Average diagrams of m6A modification scores around bases attached to chromatin (peaks) from histone H3 (B) and all chemical modification specific (C) PIRCh-seq profiles, versus that from randomly selected background.
