# Supplementary material for: PIRCh-seq: functional classification of non-coding RNAs associated with distinct histone modifications
Source: Genome Biol. 2019 Dec 20;20:292. doi: 10.1186/s13059-019-1880-3 (PMC6924075; doi:10.1186/s13059-019-1880-3)
Supplement: Supplementary file 4 — Additional file 4. Review history. [file 13059_2019_1880_MOESM4_ESM.docx]

Review History

**First round of review**

**Reviewer 1**

**Were you able to assess all statistics in the manuscript, including the appropriateness of statistical tests used?**

Yes, and I have assessed the statistics in my report.

**Were you able to directly test the methods?**

No

**Comments to author:**

The authors describe a technique (PIRCh-seq) to purify and identify RNAs associated with chromatin marked with certain histone modifications and apply it to different cell types. They find that ncRNAs overall are more likely to be associated with chromatin and that they have distinct preferences for different histone marks. They validate the technique showing that known lncRNAs behave as expected, for example XIST is enriched on chromatin marked by K27me3 and 7SK at chromatin marked by H3K4me1/3 and H3K27ac. They use a chromHMM-like strategy to classify chromatin-bound lncRNAs based on the combination of histone marks they associate and find they cluster in distinct groups with potential functional implications. They cross-analyze the signal from PIRCh-seq with icSHAPE and m6A mapping and report a tendency for chromatin-associated RNA regions to be single stranded. Finally, they report allele specific differences in Gas5 binding to chromatin caused by SNPs that affect the lncRNA structure.

This is an interesting dataset and the accompanying analyses are rigorous and at times creative. Given the bulk nature of this approach and the broad presence of histone marks on chromatin the conclusions that the authors can draw are limited, but they seem aware of this and are prudent in what they state in the results and discussion. Overall, I am supportive of publication but there are a few issues that I think the authors should address.

Major points

1. Comparisons with previous work

1.1: A similar approach has been described previously and used to profile RNAs bound to chromatin containing H4K20me3 and H3K4me3 marks in ESCs (Kurup et al. JBC 2018; PMID 30115682). This study should be acknowledged and since one of the marks is the same, the data should be compared. Also given that the technique has already a name (CARIP-seq) I am not sure the authors are justified in giving their approach a different one.

1.2: Why does Fig. 2 only use GRID as comparison for genome-wide RNA-DNA interactions and not also MARGI and SPRITE, which are mentioned in the introduction?

1.3: Why do the authors believe their method recover less nascent RNA?

1.4: In Fig. 2C, I find it odd that the low recovery of intronic reads is also true for the input. In this case it should be total nuclear RNA and match the SNE + CPE of the previous plot, that is, there should be more intronic reads than in the pull-down. How do the authors explain this?

2. Cis vs. trans

2.1: The analyses in Fig. 3F and S5 are insufficient to support the conclusions in line 383-6 that "chromatin-associated ncRNAs may function through a combinational pattern of the histone modifications" or "that the chromatin associated ncRNAs may function in cis in general". The PIRCh-protocol is a bulk analysis and these conclusions stretch the data beyond what is reasonable. In fact, they already acknowledge this limitation in the discussion (lines 569-70). To prove this point the authors would need to show by ChIRP and ChIP that the relevant lncRNAs are actually bound to the genes in the neighboring regions, and that the neighboring regions contain the relevant combination of histone marks.

2.2: In lines 210-1 and elsewhere a point is made that "functional chromatin-associated ncRNAs" and "nascent RNAs co-transcriptionally tethered to chromatin" are mutually exclusive subsets but that does not need be the case. Some lncRNAs might very well function co-transcriptionally. I think the authors should rephrase to distinguish between trans-acting lncRNas and nascent RNAs.

2.3: are the citations used on line 369 the correct ones for "may regulate gene expression in cis"?

3. Effects of differentiation

3.1: Fig. 4B is somewhat problematic as it suggest that the same lncRNAs associate with different chromatin types in different cells, if I read it correctly. Perhaps this is a function of big differences in expression levels across cell types. Would it help to only consider lncRNas that are consistently recovered at high levels in all cell types? Either way the authors should include some discussion of this observation.

3.2: The result shown in Fig. 4E is also difficult to interpret. I would understand if a ncRNA is associated with both K4me3 and K27me3 in mESCs and then only one of the two upon differentiation, but this shows that the specificity of the ncRNA "resolves" during differentiation in both direction. It is hard to reconcile this with a molecular model. ChIRP-seq for this particular ncRNA in the various cells states would help understand how this result comes about.

4. Structural conclusions.

4.1: The correlation with icSHAPE and m6A distribution is used to make the conclusion that single-stranded regions are more likely to interact with chromatin, but what if single-stranded regions are just easier to crosslink?

4.2: In Fig. 5A, the profile of the H3 and H3K4me3 PIRCh mirrors the profile of the input, suggesting that the peaks are due to clonability or other factors rather than actual domain-specific interactions. Is this the case for all RNAs? I would suggest to focus on RNA regions that are differentially enriched (i.e. peaks) compared to control.

5. Nature of interaction with chromatin. At line 464 and elsewhere the authors seem to propose that all the RNAs recovered by PIRCh-seq directly interact with histone modification. This is reading too much in the data. Given the crosslinking regimen I believe that indirect interactions to DNA or other proteins might contribute to recovery. Overall I think the authors uncover lncRNAs with preference for certain chromatin "domains", which is certainly of interest but should be stated as so. To conclude that the lncRNAs interact directly with histone modifications would require in vitro validations that I doubt the authors would want to perform for this study.

Minor points

- Fig. S1E: this is either a strange way to show similarity or I don't understand it. Are the axis properly labeled? Shoulldn't it be RPKM on the x and density on the y? Why not use a correlation heatmap?

- Fig. S3: please label axes

- Lines 237-8: rephrase to "recovery of nascent transcripts"

- Fig. S5A: please add axis label

- Fig. 3F: typo on axis label

- Fig. S6D-E: I don't understand how these support the conclusion that "MEF enriched ncRNAs are predominantly similar to those from mESC"

**Reviewer 2**

**Were you able to assess all statistics in the manuscript, including the appropriateness of statistical tests used?**

Yes, and I have assessed the statistics in my report.

**Were you able to directly test the methods?**

No

**Comments to author:**

The manuscript by Fang & Ma, et al. describes PIRCh-seq, an optimized method for identifying RNAs associated with histones containing various histone modifications. The authors use PIRCh-seq to profile multiple histone modifications in human and mouse cell lines, and identify both individual noncoding RNAs that show specific patterns of association with active versus silent chromatin states, as well as general principles of RNA association with histones that suggests convergence between noncoding RNA association and bivalent promoter states in undifferentiated cells.

Overall, I find the method well-validated and analysis insightful, and I believe this method in general as well as the datasets described here specifically will be highly useful in studying the role of noncoding RNAs in regulating chromatin state and function.

I have only two main concerns with the current manuscript:

1) The authors are convincing in showing why PIRCh-seq is better suited to the identification of histone-associated (particularly modification-specific) RNAs than the broader GRID-seq/etc unbiased RNA:DNA interaction approaches, and while I suspect CLIP-based methods could be adapted to map histone-associated RNAs I am unaware of any such effort to date. However, an approach that appears similar to PIRCh-seq, CARIP-seq, was recently used to profile H4K20me3 and H3K4me3-associated RNAs in mouse ES cells (Kurup & Kidder et al. 2018 (JBC) PMID 30115682). Particularly as H3K4me3 overlaps between the two efforts, it would be good to broadly address whether (1) PIRCh-seq shows similar RNA enrichments (and, if not, why these methods show distinct patterns), and (2) whether PIRCh-seq gives improved signal-to-noise due to improvements in this method.

2) My other main textual issue is that figures (and legends) are often not specific in defining exactly what is being shown. For example, values are often just described as 'normalized coverage', which is sometimes clear from the methods but often times unclear. These should be clarified throughout (I've noted specific particularly confusing ones below).

Specific comments:

Figure S1E - I find both the text related to this figure and the figure itself confusing. What is the x-axis (text seems to imply something like log(expression))? Is the argument just that there's a more similar distribution of 'expression'? It seems like this would be more clearly represented by simply showing scatter plots &/or calculating correlation between expression values.

Figure S1F-M - What do these plots look like when comparing, say, H3K27ac vs H3K27me3? The R values look good, but it would be helpful to have a reference to how they compare to non-same modification datasets (or whether the data overall is just highly correlated).

Figure 1b/c (& S2A) - The figures show browser tracks for the PIRCh-seq data and qPCR overall enrichment; it should be shown (or stated) the overall enrichment from the PIRCh-seq in Fig 1b & S2A. Also (unless I missed this) it should be stated explicitly somewhere what the normalized read density shown is (RPM?), and qPCR details (number of replicates, error bars, etc).

Figure 1d - Because the input is so low throughout this region it shrinks the visible dynamic range - can the authors confirm that the boxed region actually has higher fold-enrichment (what is the fold-enrichment in PIRCh versus input for the boxed region vs all other regions)? It's impossible to see whether the input in that region is, say, 100 (versus 5 everywhere else), which could mean that the fold-enrichment is not as dramatic as the read density seems to imply.

Figures 2B/C - x-axis labels should be rotated to be more clearly associated with specific positions (it seems that intron start & end / stop should be the two center tick marks, but this is unclear)

Figure S4c - inner circle is described as 'expressed' - is this just input (with some expression cutoff)?

Fig 2J - The figure shows H3K4me3 as an outgroup, not as particularly closer to the other enhancer-like states than the repressive modifications as the text suggests - this should either be shown in figure or changed in the text.

Figure 3E (& S5b,d) - 'enhancer' is misspelled

Figure 3E - The conclusion is overly non-specific: 'Overall, we partially recapitulated the chromatin classifications based on chromHMM algorithm to ChIP-seq profiles' - it would be helpful to list which classifications the authors believe were correct or missing. Is there any thoughts on why the PIRCh approach might be more sensitive to recovering certain types of chromatin states than others?

Figure 3F - The change in mean (dotted line) is extremely difficult to see in this plot - perhaps they can be replotted as a sub-figure with a smaller y-axis range? The boxplots obscure the text-stated difference between means comparing across 3F and S5c.

Figure S6F - the text seems to suggest a different figure here (showing enrichment in IgG for PIRCh-enriched ncRNAs)?

Fig 5B / 422-424 - this conclusion is strangely worded, since the simple interpretation of the results would be that there is simply less specificity in histone:RNA complexes in mESCs, not that specificity is more 'plastic' (I'm unclear what that would mean).

Fig 5C - Is there a way to show that that this couldn't simply reflect some overall experimental issue in mESCs (better yield, better IP success, deeper sequencing, etc)?

Fig 5G - It seems surprising that if 129S1 is the only allele expressed (as the authors reference), that Xist would have only a 2-fold increase between 129S1 vs CAST in both IP and input. Do the authors think this reflects the underlying limitation in signal to noise in this analysis, or does the data here suggest that CAST Xist is actually expressed as well?

Methods:

As sonication is likely a significant factor in specificity, the authors should provide details on sonicator equipment and conditions/timing.

Lines 160-161 "We found a total of 23 expressed lncRNAs in the LnChrom database, including Xist, Firre, Rmrp, Tug1 and etc.," - the list of 23 would be helpful to provide as a list here or supplementary table

Lines 220-222 "Finally, we calculated the relative fold-change of each peak with respect to the input control. Significant peaks were filtered based on fold change and P-value." - what statistical test was used to determine p-value?

**Authors Response**

**Point-by-point responses to the reviewers’ comments:**

Responses to reviewers

We thank the reviewers for their positive assessments and thoughtful suggestions in improving this work. Point-by-point responses are as follows.

We have revised our manuscript mainly in the following aspects:

1.	Ensure the manuscript conforms to meet the style of Method article style, including shortened the Abstracts to meet the words limitation, add Conclusions section and merge Methods section to manuscript.

2.	Compared PIRCh-seq with other state-of-the-art methods used to profile chromatin associated RNAs, including MARGI and SPRINT as the reviewers suggested. However, we were not able to obtain the raw sequencing data nor the chromatin enriched gene lists from CARIP-seq, and therefore unable to perform a comparison. The authors of the CARIP paper did not upload their data to GEO or any public repository, and did not share the original data despite three requests.

3.	We rephrased several sentences and made the manuscript more precise. Meanwhile revised figures and figure legends to clarify ambiguous points, including Fig. 1B-F, Fig. 2B-C, Fig. 3F, and Fig. 5A and a few supplemental figures as well.

Reviewer #1 (Remarks to the Author):

The authors describe a technique (PIRCh-seq) to purify and identify RNAs associated with chromatin marked with certain histone modifications and apply it to different cell types. They find that ncRNAs overall are more likely to be associated with chromatin and that they have distinct preferences for different histone marks. They validate the technique showing that known lncRNAs behave as expected, for example XIST is enriched on chromatin marked by K27me3 and 7SK at chromatin marked by H3K4me1/3 and H3K27ac. They use a chromHMM-like strategy to classify chromatin-bound lncRNAs based on the combination of histone marks they associate and find they cluster in distinct groups with potential functional implications. They cross-analyze the signal from PIRCh-seq with icSHAPE and m6A mapping and report a tendency for chromatin-associated RNA regions to be single stranded. Finally, they report allele specific differences in Gas5 binding to chromatin caused by SNPs that affect the lncRNA structure.

This is an interesting dataset and the accompanying analyses are rigorous and at times creative. Given the bulk nature of this approach and the broad presence of histone marks on chromatin the conclusions that the authors can draw are limited, but they seem aware of this and are prudent in what they state in the results and discussion. Overall, I am supportive of publication but there are a few issues that I think the authors should address.

*Response: We thank the reviewer for their positive assessment that our dataset is interesting and the accompanying analyses are rigorous, as well as for their supports for publication of our manuscript.*

Major points:

1. Comparisons with previous work

1.1: A similar approach has been described previously and used to profile RNAs bound to chromatin containing H4K20me3 and H3K4me3 marks in ESCs (Kurup et al. JBC 2018; PMID 30115682). This study should be acknowledged and since one of the marks is the same, the data should be compared. Also given that the technique has already a name (CARIP-seq) I am not sure the authors are justified in giving their approach a different one.

*Response: We thank the reviewer’s suggestion and immediately tried to download data from the CARIP-seq paper (Kurup et al. JBC 2018)[1]. However, after carefully read through this published paper, we noticed that neither the raw sequencing profile nor the chromatin enriched gene lists were included in the original paper or uploaded to any public online databases, despite the fact that this paper was published nearly 1 year ago. We then contacted the authors of the CARIP-seq paper via several emails requesting for sequencing data. We were told that the authors did plan to submit the data to a public repository such as NCBI GEO in future, but they did not share the data with us.*

*Again, we appreciated the reviewer’s suggestion by adding the CARIP-seq paper as a reference of recent approach. However, since we do not have the raw CARIP-seq data nor the chromatin enriched gene lists in hand, we were not able to make any quantitative comparison of the results from CARIP-seq and our PIRCh-seq.*

*In addition, PIRCh-seq is different from CARIP-seq in the following two aspects:*

*1)	The PIRCh approach was enabled by our recent development of robust methods, named ChIRP-seq (Chu et al. Molecular Cell)[2] and HiChIRP (Mumbach et al. Nature Methods 2019)[3] to crosslink endogenous RNA-chromatin interactions in living cells using glutaraldehyde as the crosslinking reagent, where we have shown that glutaraldehyde was very efficient in capturing RNA-chromatin interactions. CARIP-seq uses a different crosslinking method, which was 1% of formaldehyde for 1 min. As tested by ChIRP-southern in [2], 1% glutaraldehyde is better than 1% formaldehye crosslinking in capturing RNA-chromatin associations. We further optimized downstream library preparation methods for deep sequencing.*

*2)	Compare to previous fRIP-seq data profiling RNAs bound to TF (Hendrickson et al. Genome Biology 2011)[4], which is similar to CARIP-seq but 0.1% of formaldehyde for 10 min , PIRCh-seq identifies associated RNAs with higher signal-to-noise ratio.*

*Therefore, we would like to keep the name of PIRCh-seq because our method has fundamental differences from CARIP-seq. There are many precedents: ChIRP-seq and RAP-seq both refer to RNA pulldown of chromatin, but ChIRP uses short oligos while RAP uses longer oligonucleotides. We are also uncomfortably calling our method CARIP-seq since we are not able to compare our results with the primary data of Kurup et al. It would make for a very confusing situation if two protocols with the same name gave very different results.*

1.2: Why does Fig. 2 only use GRID as comparison for genome-wide RNA-DNA interactions and not also MARGI and SPRITE, which are mentioned in the introduction?

*Response: MARGI uses similar experimental method as GRID-seq to capture chromatin associated RNAs, whose comparison was included in the revised Figure 2. As expected, we found that the read counts distribution of MARGI was also very close to that from GRID. Since Figure 2 was performed based on data from human cell lines, we supplemented our comparison between PIRCh and SPRITE in V6.5 ES cells as follows, and we found:*

*1)	PIRCh-seq shows slightly better signal-to-noise ratio than SPRITE in V6.5 ES cell line (see below left).*

*2)	However, as was also shown in the SPRITE paper, only 1% of the total sequencing reads were mapped to RNAs (Table S6B in Quinodoz et al. Cell 2018)[5], of which 70% were rRNAs, making SPRITE-seq not suitable to profile chromatin-associated ncRNAs. On the contrary, the rRNAs consist only 20% to 30% of reads in PIRCh-seq (see above right).*

*These results suggest that PIRCh-seq is better than SPRITE in capturing chromatin associated ncRNAs.*

1.3: Why do the authors believe their method recover less nascent RNA?

*Response: Nascent RNAs are still captured by PIRCh. Some level of nascent transcription can still be visualized using PIRCh-seq; however, it is relatively low compared to other methods capturing RNA-chromatin interaction (Figure 2A-C, Figure S3A-E). One likely explanation is that glutaraldehyde is a longer span crosslinking reagent (5 carbons instead of 1 carbon in formaldehyde) that is capable of capturing more regulatory RNAs which are able to interact over a longer range compared to nascent transcripts (Hsieh et al. Nature Methods, 2016) [6] (Zeng et al. Biotechniques 2006) [7]. To estimate the level of nascent transcription in PIRCh, we calculated the average read counts around the spliced introns. We found that the reads on introns were significantly lower in PIRCh, compared with similar methods, such as GRID-seq, MARGI, and other traditional chromatin-binding RNA detection technologies like soluble-nuclear extract (SNE), chromatin pellet extract (CPE) and chromatin-associated RNAs (CAR) (Figure 2A-B). To further estimate the level of nascent transcription, we then integrated each histone modification-specific PIRCh-seq profile with its corresponding ChIP-seq dataset from the same cell line - in this case the V6.5 mESC - and asked whether the PIRCh-seq signal of each RNA correlated with the nearby ChIP-seq signal of the corresponding modification (see Methods). The ChIP-seq profiles of each histone modification in mESC were obtained from ENCODE, and our results indicated that there was no significant correlation with these two sets of signals (Figure S3A-E, R~0). These results suggest that the majority of PIRCh-seq enriched chromatin-associated RNAs are mature RNAs with introns spliced out, and the percentage of nascent transcription from PIRCh-seq is negligible.*

1.4: In Fig. 2C, I find it odd that the low recovery of intronic reads is also true for the input. In this case it should be total nuclear RNA and match the SNE + CPE of the previous plot, that is, there should be more intronic reads than in the pull-down. How do the authors explain this?

*Response: Instead of total nuclear RNA, the “input” used for PIRCh enrichment analysis was the lysate we took after crosslinking and sonication right before IP. So input is more like total RNA with mature mRNA which may contribute to lower intron ratio. Since IP will enrich chromatin fraction, there should be more nascent transcript in IP group than “input”.*

2. Cis vs. trans

2.1: The analyses in Fig. 3F and S5 are insufficient to support the conclusions in line 383-6 that "chromatin-associated ncRNAs may function through a combinational pattern of the histone modifications" or "that the chromatin associated ncRNAs may function in cis in general". The PIRCh-protocol is a bulk analysis and these conclusions stretch the data beyond what is reasonable. In fact, they already acknowledge this limitation in the discussion (lines 569-70). To prove this point the authors would need to show by ChIRP and ChIP that the relevant lncRNAs are actually bound to the genes in the neighboring regions, and that the neighboring regions contain the relevant combination of histone marks.

*Response: We agree with the reviewer that further investigations are still required to conclude whether chromatin-associated ncRNAs are functioning in cis or trans, and the combinational pattern of chromatin association may be caused by the bulk nature of PIRCh-seq analysis. Therefore, we removed this sentence “These results not only indicate that the chromatin-associated ncRNAs may function through a combinational pattern of the histone modifications instead of an individual modification, but also favors the argument that the chromatin associated ncRNAs may function in cis in general” from the manuscript and added “However further investigations using ChIRP and ChIP analysis, preferable at single cell level are still required to fully uncover their regulatory patterns on chromatin.”*

2.2: In lines 210-1 and elsewhere a point is made that "functional chromatin-associated ncRNAs" and "nascent RNAs co-transcriptionally tethered to chromatin" are mutually exclusive subsets but that does not need be the case. Some lncRNAs might very well function co-transcriptionally. I think the authors should rephrase to distinguish between trans-acting lncRNas and nascent RNAs.

*Response: We thank the reviewer’s suggestion and rephrased the sentence to “Furthermore, proximity ligation methods predominantly detect nascent RNAs co-transcriptionally tethered to chromatin by RNA polymerase, confounding background signal from all RNAs in the process of transcription”. We also double checked the entire manuscript to make sure that "functional chromatin-associated ncRNAs" and "nascent RNAs co-transcriptionally tethered to chromatin" are not stated as if they are mutually exclusive.*

2.3: are the citations used on line 369 the correct ones for "may regulate gene expression in cis"?

*Response: That was indeed a mis-citation. We also rephrased the sentence in the main text and replaced two references on cis or trans regulation of lncRNA(Yan et al. Current Opinion in Genetics & Development 2017)(Kopp et al. Cell 2018)[8,9].*

3. Effects of differentiation

3.1: Fig. 4B is somewhat problematic as it suggests that the same lncRNAs associate with different chromatin types in different cells, if I read it correctly. Perhaps this is a function of big differences in expression levels across cell types. Would it help to only consider lncRNas that are consistently recovered at high levels in all cell types? Either way the authors should include some discussion of this observation.

*Response: Fig. 4B was obtained from the enrichment scores of all the expressed (filtered by edgeR package which is recommended by limma) and also chromatin enriched lncRNAs (458 in total) in any of the three cell types. For each lncRNA, its enrichment score was defined as the PIRCh signal over input, and therefore, the expression differences of the same lncRNA in different cells were normalized. To further reduce the impact of the expression divergence between cell types, we plotted a same figure with 382 ncRNAs that were expressed (RPKM>1, see below left) in all of 3 cell types, from which we still saw 3 distinct clusters of chromatin enrichments by cell types (see below). For higher expressed (RPKM > 10, n=110, see below right), we can still see the difference of cell types dominate the cluster except H3K27me3 in MEF. In addition, we added this sentence “where the expression divergence of the same lncRNA in different cells were normalized” in the main text.*

RPKM>1, n=382	RPKM>10, n=110

3.2: The result shown in Fig. 4E is also difficult to interpret. I would understand if a ncRNA is associated with both K4me3 and K27me3 in mESCs and then only one of the two upon differentiation, but this shows that the specificity of the ncRNA "resolves" during differentiation in both directions. It is hard to reconcile this with a molecular model. ChIRP-seq for this particular ncRNA in the various cells states would help understand how this result comes about.

*Response: ES cells are known to be enriched for bivalent chromatin, comprised of both H3K4me3 and H3K4me27 on the same nucleosomes (Mikkelsen et al. Nature 2007) (Voigt et al. Cell 2012)[10,11] that mark “poised” developmental genes that may either turn on (marked by H3K4me3 only) or turn off (marked by H3K27me3 only) in distinct differentiated cell types. We interpret our PIRCh-seq result to mean that certain ncRNAs can associate with H3K27me3 or H3K4me3 in either the bivalent or monovalent state. The specificity of the ncRNA has not necessarily changed, but we are observing the combinatorial action of the histone code in cell differentiation. We have cited the above references and clarified our interpretation in the text.*

4. Structural conclusions.

4.1: The correlation with icSHAPE and m6A distribution is used to make the conclusion that single-stranded regions are more likely to interact with chromatin, but what if single-stranded regions are just easier to crosslink?

*Response: Crosslinking used by glutaraldehyde and formaldehyde has been widely applied to crosslink doubles-stranded nucleic acid, e.g DNA in ChIP (Ren et al. Science 2000) [12](lyer et al. Nature 2001) [13](Mikkelsen et al. Cell 2017) [10], and RNA in ChIRP(Chu et al. Molecular Cell 2011)[2], RIP(Zhao et al. Molecular Cell 2011)[14] with no reported structural bias. For example, RIP and fRIP-seq were applied to investigate dsRNA binding to MLE (Lv et al, Nucleic Acids Research 2019)[15] or MDA5(Zhao et al. Nature Communication 2018)[16], and previous paper recommended using formaldehyde instead of UV for RIP to be less biased[17]. Recent publication(Dueva et al, Cell Chemical Biology 2019)[18] on RNA-chromatin interaction also suggested that single-strand RNAs can more effectively prevent precipitation of histones and can attenuate electrostatic interactions between histones and DNA, thereby loosening up the chromatin structure. These findings support our discovery that single-strand RNAs are more likely to interact with chromatin from PIRCh-seq*.

4.2: In Fig. 5A, the profile of the H3 and H3K4me3 PIRCh mirrors the profile of the input, suggesting that the peaks are due to clonability or other factors rather than actual domain-specific interactions. Is this the case for all RNAs? I would suggest to focus on RNA regions that are differentially enriched (i.e. peaks) compared to control.

*Response: We thank the reviewer’s suggestion. In fact, that was exactly what we had done. The enriched domain specific interactions were obtained from a sliding window based peak calling method and filtered by P-value and fold change over control (see Methods). To better illustrate these domain specific peaks, we plotted additional curves on top of Figure 1D and Figure 5A representing the fold enrichments of the PIRCh-seq signal over inputs.*

5. Nature of interaction with chromatin. At line 464 and elsewhere the authors seem to propose that all the RNAs recovered by PIRCh-seq directly interact with histone modification. This is reading too much in the data. Given the crosslinking regimen I believe that indirect interactions to DNA or other proteins might contribute to recovery. Overall I think the authors uncover lncRNAs with preference for certain chromatin "domains", which is certainly of interest but should be stated as so. To conclude that the lncRNAs interact directly with histone modifications would require in vitro validations that I doubt the authors would want to perform for this study.

*Response: We thank the reviewer’s suggestion and agree that PIRCh-seq alone is not sufficient to distinguish “direct” or “indirect” interactions between lncRNAs and chromatin. Thereby we have modified all the relevant sentences about direct/indirect interaction.*

Minor points

- Fig. S1E: this is either a strange way to show similarity or I don't understand it. Are the axis properly labeled? Shoulldn't it be RPKM on the x and density on the y? Why not use a correlation heatmap?

*Response: We’ve changed to correlation heatmap as suggested.*

- Fig. S3: please label axes

*Response: Corrected in the revised manuscript, thanks.*

- Lines 237-8: rephrase to "recovery of nascent transcripts"

*Response: Corrected in the revised manuscript, thanks.*

- Fig. S5A: please add axis label

*Response: Corrected in the revised manuscript, thanks.*

- Fig. 3F: typo on axis label

*Response: Corrected in the revised manuscript, thanks.*

- Fig. S6D-E: I don't understand how these support the conclusion that "MEF enriched ncRNAs are predominantly similar to those from mESC"

*Response: We rephrased the sentence to “The chromatin enriched ncRNAs in MEF also form a combinatorial pattern with multiple histone modifications”.*

Reviewer #2 (Remarks to the Author):

The manuscript by Fang & Ma, et al. describes PIRCh-seq, an optimized method for identifying RNAs associated with histones containing various histone modifications. The authors use PIRCh-seq to profile multiple histone modifications in human and mouse cell lines, and identify both individual noncoding RNAs that show specific patterns of association with active versus silent chromatin states, as well as general principles of RNA association with histones that suggests convergence between noncoding RNA association and bivalent promoter states in undifferentiated cells.

Overall, I find the method well-validated and analysis insightful, and I believe this method in general as well as the datasets described here specifically will be highly useful in studying the role of noncoding RNAs in regulating chromatin state and function.

*Response: We thank the reviewer for their positive assessment as well as for their constructive suggestions to improve the manuscript.*

I have only two main concerns with the current manuscript:

1) The authors are convincing in showing why PIRCh-seq is better suited to the identification of histone-associated (particularly modification-specific) RNAs than the broader GRID-seq/etc unbiased RNA:DNA interaction approaches, and while I suspect CLIP-based methods could be adapted to map histone-associated RNAs I am unaware of any such effort to date. However, an approach that appears similar to PIRCh-seq, CARIP-seq, was recently used to profile H4K20me3 and H3K4me3-associated RNAs in mouse ES cells (Kurup & Kidder et al. 2018 (JBC) PMID 30115682). Particularly as H3K4me3 overlaps between the two efforts, it would be good to broadly address whether (1) PIRCh-seq shows similar RNA enrichments (and, if not, why these methods show distinct patterns), and (2) whether PIRCh-seq gives improved signal-to-noise due to improvements in this method.

*Response: As we indicated above to Reviewer 1 Question 1.1, we would love to compare PIRCh-seq with the CARIP-seq results. However, because the authors did not release the raw CARIP-seq data nor the chromatin enriched gene lists, we were not able to perform the comparison of the results from these two methods. We are left in a frustrating position of citing Kurup et al. without being able to comment on relative performance. In addition, there are multiple differences between PIRCh and CARIP, as we explained in response to Reviewer 1 Question 1.1.*

2) My other main textual issue is that figures (and legends) are often not specific in defining exactly what is being shown. For example, values are often just described as 'normalized coverage', which is sometimes clear from the methods but often times unclear. These should be clarified throughout (I've noted specific particularly confusing ones below).

*Response: We apologize to confuse the reviewer and potential readers as well, and have revised figures and figure legends to clarify ambiguous places. We appreciate the reviewers’ detailed suggestions.*

Figure S1E - I find both the text related to this figure and the figure itself confusing. What is the x-axis (text seems to imply something like log(expression))? Is the argument just that there's a more similar distribution of 'expression'? It seems like this would be more clearly represented by simply showing scatter plots &/or calculating correlation between expression values.

*Response: We’ve changed Figure S1E to correlation heatmap. Our result shows that the expressions of the input RNAs are similar to that of the total RNAs, and then from the nuclear extraction and least similar to cytoplasm.*

Figure S1F-M - What do these plots look like when comparing, say, H3K27ac vs H3K27me3? The R values look good, but it would be helpful to have a reference to how they compare to non-same modification datasets (or whether the data overall is just highly correlated).

*Response: Figure S1F-M were used to illustrate the high reproducibility of the PIRCh-seq experiments, and we did not intend to compare non-same modification datasets in the previous submission. We now show the correlation heatmap between all samples below, from which we can see that the data overall is highly correlated. This is because there are very few (in our case 258 in mESC) chromatin enriched ncRNAs compare to tens of thousands RNAs in a cell. These chromatin enriched ncRNAs were also attached to places with multiple histone modifications, and therefore the differences between non-same modification datasets were submerged in random fluctuations.*

Figure 1b/c (& S2A) - The figures show browser tracks for the PIRCh-seq data and qPCR overall enrichment; it should be shown (or stated) the overall enrichment from the PIRCh-seq in Fig 1b & S2A. Also (unless I missed this) it should be stated explicitly somewhere what the normalized read density shown is (RPM?), and qPCR details (number of replicates, error bars, etc).

*Response: We have revised the figures by adding labeling axes. We also updated figure legends to make it clearer for the readers by adding “the overall enrichment of the H3 PIRCh-seq signal over input” for Figure 1B & S2A. Unfortunately, no replicates were performed for the qPCR experiment in Figure 1C.*

Figure 1d - Because the input is so low throughout this region it shrinks the visible dynamic range - can the authors confirm that the boxed region actually has higher fold-enrichment (what is the fold-enrichment in PIRCh versus input for the boxed region vs all other regions)? It's impossible to see whether the input in that region is, say, 100 (versus 5 everywhere else), which could mean that the fold-enrichment is not as dramatic as the read density seems to imply.

*Response: We thank for the reviewer’s suggestion and revised Figure 1D by providing a sliding-window based fold-enrichment of PIRCh over input along the transcript. We can see that the boxed region is one of the most highly enriched regions on the Xist transcript, and signal in H3K27me3 PIRCh is higher than those in H3K4me3 and H3K27ac PIRCh experiments.*

Figures 2B/C - x-axis labels should be rotated to be more clearly associated with specific positions (it seems that intron start & end / stop should be the two center tick marks, but this is unclear)

*Response: Corrected in the revised manuscript, thanks.*

Figure S4c - inner circle is described as 'expressed' - is this just input (with some expression cutoff)?

*Response: Yes, and the low-expressed genes were filtered by the “filterByExpr” module in edgeR (see Methods) as the limma package recommended. We revised the legend for Figure S4C to illustrate this point.*

Fig 2J - The figure shows H3K4me3 as an outgroup, not as particularly closer to the other enhancer-like states than the repressive modifications as the text suggests - this should either be shown in figure or changed in the text.

*Response: Corrected in the revised manuscript, thanks.*

Figure 3E (& S5b,d) - 'enhancer' is misspelled

*Response: Corrected in the revised Figures, thanks.*

Figure 3E - The conclusion is overly non-specific: 'Overall, we partially recapitulated the chromatin classifications based on chromHMM algorithm to ChIP-seq profiles' - it would be helpful to list which classifications the authors believe were correct or missing. Is there any thoughts on why the PIRCh approach might be more sensitive to recovering certain types of chromatin states than others?

*Response: The chromHMM algorithm was widely applied to classify chromatin states based on a combinational histone modification ChIP-seq profiles(Ernst et al. Nature 2011)[19], and usually more different types of ChIP-seq datasets included, more precise the chromatin states classification can be reached. Similarly, since PIRCh-seq is capable to reveal histone modification specific chromatin association in high precision, we believe that more histone modification specific PIRCh-seq enrolled more precise the chromatin states can be predicted. However, since we only included 6 most widely used antibodies this study, namely H3K4me1, H3K4me3, H3K27ac, H3K27me3, H3K9me3 and H4K16ac, the classification of chromatin states was incomplete. We rephrased the sentence to “Overall, we partially recapitulated the chromatin classifications by applying the chromHMM algorithm based on PIRCh-seq profiles with 6 well studied histone modifications” to make it more specific. On the other hand, since other methods were not capable to detecting histone modification specific chromatin association, may not as sensitive as PIRCh-seq to recovering or distinguishing certain types of chromatin states.*

Figure 3F - The change in mean (dotted line) is extremely difficult to see in this plot - perhaps they can be replotted as a sub-figure with a smaller y-axis range? The boxplots obscure the text-stated difference between means comparing across 3F and S5c.

*Response: We’ve reduced the range of y-axis from 11 to 4 for both Figure 3F and S5C in the revised manuscript so that the change in mean can be better seen.*

Figure S6F - the text seems to suggest a different figure here (showing enrichment in IgG for PIRCh-enriched ncRNAs)?

*Response: Corrected in the revised manuscript, thanks.*

Fig 5B / 422-424 - this conclusion is strangely worded, since the simple interpretation of the results would be that there is simply less specificity in histone:RNA complexes in mESCs, not that specificity is more 'plastic' (I'm unclear what that would mean).

*Response: We think the reviewer was actually talking about Figure 4B, instead of Figure 5B in lines 422-424. We have rephrased the sentence to “Interestingly, compared with those of the more differentiated cells (MEF and NPC), the ncRNA chromatin association in mESCs showed a higher correlation coefficient among distinct histone modifications, suggesting less specificity in chromatin-ncRNAs association in mESCs than those in differentiated cells (Figure 4B).”*

Fig 5C - Is there a way to show that that this couldn't simply reflect some overall experimental issue in mESCs (better yield, better IP success, deeper sequencing, etc)?

*Response: We are not sure whether the reviewer was asking about Figure 5C or Figure 4C. We chose V6.5 mESC as an example cell line to study the relationship between RNA structure and chromatin association because both RNA structure profiles from icSHAPE and RNA-chromatin interaction dataset from PIRCh-seq were available. However, if the reviewer was asking about Figure 4C, then to our knowledge, we believe there was no systematic experimental issue in mESC. We used the same set of antibodies, with exactly the same protocol in both experimentally an analytically. In addition, we performed replicates on each sample and applied a rigorous method to extract the significant enriched ncRNAs, where the yield, IP efficiency, as well as sequencing depth were all normalized (see Methods). On the other hand, if there was an overall experiment bias in mESC (or other cell types), we were supposed to see monotonically more (or less) chromatin associated ncRNAs in every histone modification in mESC (or other cell types), which was not what we have discovered in Figure 4C.*

Fig 5G - It seems surprising that if 129S1 is the only allele expressed (as the authors reference), that Xist would have only a 2-fold increase between 129S1 vs CAST in both IP and input. Do the authors think this reflects the underlying limitation in signal to noise in this analysis, or does the data here suggest that CAST Xist is actually expressed as well?

*Response: We apologize that we performed double log2 (fold change) in our previous submission, and this mistake has been corrected in this revision. Thereby the actual fold increase of 129S1 VS CAST was over 16. The detailed allele-specific analysis results were included in SuppTable2. Thank you for catching this error.*

Methods:

As sonication is likely a significant factor in specificity, the authors should provide details on sonicator equipment and conditions/timing.

*Response: The sonication and timing information has been updated in the Methods section. “For sonication, we used the Covaris E220 equipment with the following settings: Fill level 10, Duty Cycle 15, PIP 140, Cycles/Burst 200. In terms of time, we sonicated 20 minute pulses to test the optimal time to generate chromatin size ~300-2000bp.”*

Lines 160-161 "We found a total of 23 expressed lncRNAs in the LnChrom database, including Xist, Firre, Rmrp, Tug1 and etc.," - the list of 23 would be helpful to provide as a list here or supplementary table

*Response: The list of those 23 lncRNAs were presented in Figure 2G.*

Lines 220-222 "Finally, we calculated the relative fold-change of each peak with respect to the input control. Significant peaks were filtered based on fold change and P-value." - what statistical test was used to determine p-value?

*Response: As we mentioned above “Next, we applied a bootstrap method by randomly sampling 1000 times with reads from the transcripts, and then estimated the P-value of each peak as the percentage of cases that were more enriched than observed”. That was how the P-value was estimated for each sliding-window.*

References:

1. Kurup JT, Kidder BL. Identification of H4K20me3- and H3K4me3-associated RNAs using CARIP-Seq expands the transcriptional and epigenetic networks of embryonic stem cells. J. Biol. Chem. American Society for Biochemistry and Molecular Biology; 2018;293:15120–35.

2. Chu C, Qu K, Zhong FL, Artandi SE, Chang HY. Genomic maps of long noncoding RNA occupancy reveal principles of RNA-chromatin interactions. Molecular Cell. 2011;44:667–78.

3. Mumbach MR, Granja JM, Flynn RA, Roake CM, Satpathy AT, Rubin AJ, et al. HiChIRP reveals RNA-associated chromosome conformation. Nat. Methods. Nature Publishing Group; 2019;16:489–92.

4. G Hendrickson D, Kelley DR, Tenen D, Bernstein B, Rinn JL. Widespread RNA binding by chromatin-associated proteins. Genome Biol. BioMed Central; 2016;17:674.

5. Quinodoz SA, Ollikainen N, Tabak B, Palla A, Schmidt JM, Detmar E, et al. Higher-Order Inter-chromosomal Hubs Shape 3D Genome Organization in the Nucleus. Cell. 2018;174:744–757.e24.

6. Hsieh T-HS, Fudenberg G, Goloborodko A, Rando OJ. Micro-C XL: assaying chromosome conformation from the nucleosome to the entire genome. Nat. Methods. Nature Publishing Group; 2016;13:1009–11.

7. Zeng P-Y, Vakoc CR, Chen Z-C, Blobel GA, Berger SL. In vivo dual cross-linking for identification of indirect DNA-associated proteins by chromatin immunoprecipitation. BioTechniques. Future Science Ltd London, UK; 2006;41:694–8.

8. Yan P, Luo S, Lu JY, Shen X. Cis- and trans-acting lncRNAs in pluripotency and reprogramming. Curr. Opin. Genet. Dev. 2017;46:170–8.

9. Kopp F, Mendell JT. Functional Classification and Experimental Dissection of Long Noncoding RNAs. Cell. 2018;172:393–407.

10. Mikkelsen TS, Ku M, Jaffe DB, Issac B, Lieberman E, Giannoukos G, et al. Genome-wide maps of chromatin state in pluripotent and lineage-committed cells. Nature. Nature Publishing Group; 2007;448:553–60.

11. Voigt P, LeRoy G, Drury WJ, Zee BM, Son J, Beck DB, et al. Asymmetrically modified nucleosomes. Cell. 2012;151:181–93.

12. Ren B, Robert F, Wyrick JJ, Aparicio O, Jennings EG, Simon I, et al. Genome-wide location and function of DNA binding proteins. Science. American Association for the Advancement of Science; 2000;290:2306–9.

13. Iyer VR, Horak CE, Scafe CS, Botstein D, Snyder M, Brown PO. Genomic binding sites of the yeast cell-cycle transcription factors SBF and MBF. Nature. Nature Publishing Group; 2001;409:533–8.

14. Zhao J, Ohsumi TK, Kung JT, Ogawa Y, Grau DJ, Sarma K, et al. Faculty of 1000 evaluation for Genome-wide identification of polycomb-associated RNAs by RIP-seq. Molecular Cell. 2011. pp. 939–53.

15. Lv M, Yao Y, Li F, Xu L, Yang L, Gong Q, et al. Structural insights reveal the specific recognition of roX RNA by the dsRNA-binding domains of the RNA helicase MLE and its indispensable role in dosage compensation in Drosophila. Nucleic Acids Res. 2019;47:3142–57.

16. Zhao Y, Ye X, Dunker W, Song Y, Karijolich J. RIG-I like receptor sensing of host RNAs facilitates the cell-intrinsic immune response to KSHV infection. Nat Commun. Nature Publishing Group; 2018;9:4841–14.

17. Singh G, Ricci EP, Moore MJ. RIPiT-Seq: a high-throughput approach for footprinting RNA:protein complexes. Methods. 2014;65:320–32.

18. Dueva R, Akopyan K, Pederiva C, Trevisan D, Dhanjal S, Lindqvist A, et al. Neutralization of the Positive Charges on Histone Tails by RNA Promotes an Open Chromatin Structure. Cell Chem Biol. 2019.

19. Ernst J, Kheradpour P, Mikkelsen TS, Shoresh N, Ward LD, Epstein CB, et al. Mapping and analysis of chromatin state dynamics in nine human cell types. Nature. Nature Research; 2011;473:43–9.

**Second round of review**

**Reviewer 1**

I am satisfied with the authors' revisions and responses and I have no further concern about this manuscript.

**Reviewer 2**

The authors have addressed my concerns and I support publication.
